# Outcomes Following Taxation of Sugar-Sweetened Beverages: A Systematic Review and Meta-analysis

**DOI:** 10.1001/jamanetworkopen.2022.15276

**Published:** 2022-06-01

**Authors:** Tatiana Andreyeva, Keith Marple, Samantha Marinello, Timothy E. Moore, Lisa M. Powell

**Affiliations:** 1 Department of Agricultural and Resource Economics, Rudd Center for Food Policy & Health, University of Connecticut, Hartford; 2 The Heller School for Social Policy and Management, Brandeis University, Waltham, Massachusetts; 3 Health Policy and Administration, School of Public Health, University of Illinois Chicago; 4 Statistical Consulting Services, Center for Open Research Resources & Equipment, University of Connecticut, Storrs

## Abstract

**Importance:**

More than 45 countries and several local jurisdictions have implemented sugar-sweetened beverage (SSB) taxes to improve nutrition and population health, and evidence on their outcomes to date is essential to inform policy discussions. Responding to this need, the World Health Organization commissioned a systematic literature review on the outcomes of fiscal policies, including SSB taxes.

**Objective:**

To assess the associations of implemented SSB taxes with prices, sales, consumption, diet, body weight, product changes, unintended consequences, health, and pregnancy outcomes.

**Data Sources:**

Searches of 8 bibliographic databases (Business Source Complete, Cochrane Central Register of Controlled Trials, Cochrane Database of Systematic Reviews, CINAHL, EconLit, PsycINFO, PubMed, and Scopus) were performed from database inception through June 1, 2020, with no language or setting restrictions. Grey literature was assessed using 14 sources and government websites.

**Study Selection:**

The review included primary studies of implemented SSB taxes.

**Data Extraction and Synthesis:**

The review followed the Preferred Reporting Items for Systematic Reviews and Meta-analyses guidelines. For prices, sales and consumption, results were meta-analyzed using a 3-level random-effects model. Study quality was assessed at the outcome level.

**Main Outcomes and Measures:**

Tax pass-through rate for prices, percentage reduction in SSB demand, and price elasticity of demand for sales and consumption. Heterogeneity was assessed using τ^2^ and the *I*^2^ statistic.

**Results:**

A total of 86 articles were eligible, with 62 studies contributing to the meta-analysis. The overall tax pass-through rate was 82% (95% CI, 66% to 98%; *P* < .001, *I*^2^ = 99%), suggesting tax undershifting. The demand for SSBs was highly sensitive to tax-induced price increases, with the price elasticity of demand of −1.59 (95% CI, −2.11 to −1.08; *P* < .001; *I*^2^ = 100%) and a mean reduction in SSB sales of 15% (95% CI, −20% to −9%; *P* < .001; *I*^2^ = 100%). There was no evidence of substitution to untaxed beverages, and changes in SSB consumption were not significant. The narrative synthesis found reformulation and reduced sugar content of taxed beverages for tiered taxes, cross-border shopping in most studies of local-level taxes, and no negative changes in employment. Data on the heterogeneity of SSB tax outcomes across subpopulations were limited.

**Conclusions and Relevance:**

In this systematic review and meta-analysis of implemented SSB taxes worldwide, SSB taxes were associated with higher prices and lower sales of taxed beverages.

## Key Points

**Question:**

What are the outcomes of implemented sugar-sweetened beverage (SSB) taxes around the world?

**Findings:**

In this systematic review of 86 studies and a meta-analysis of 62 studies, implemented SSB taxes were associated with higher prices of targeted beverages (tax pass-through of 82%) and 15% lower SSB sales, with a price elasticity of demand of −1.59. No negative changes in employment were identified.

**Meaning:**

These findings suggest that SSB taxes may work as intended in reducing demand for SSBs through higher prices, yet further research is needed to understand their associations with diet and health outcomes and heterogeneity of consumer responses.

## Teaser

This systematic review and meta-analysis assesses the associations of implemented SSB taxes with prices, sales, consumption, diet, body weight, product changes, unintended consequences, health, and pregnancy outcomes.

## Introduction

Sugar-sweetened beverage (SSB) taxes are proposed as a policy tool to address the increasing prevalence of poor diet, obesity, and related economic and social costs.^1,2^ Noncommunicable diseases (NCDs) account for 71% of deaths globally, of which an estimated 40% could be attributed to dietary factors.^3,4^ Recently, concerns about diet-related NCDs grew further because of their association with more severe clinical outcomes from COVID-19, including hospitalization and death.^5,6^ There are well-documented negative health consequences of excessive SSB consumption in children and adults, including weight gain and increased risk of type 2 diabetes, cardiovascular disease, dental caries, and osteoporosis.^7,8,9^

To improve nutrition and health and to raise revenue, various types of SSB taxes have been implemented in more than 45 countries, including numerous subnational local jurisdictions.^10^ Evidence on their effects is growing as multiple evaluations are undertaken to provide policy makers with comprehensive real-time data. Prior systematic reviews^11,12,13,14,15,16,17^ suggested that price interventions and fiscal policies targeting SSBs and other unhealthy products could influence consumer choices and reduce demand. Much of this earlier literature was based on price data and simulation studies owing to the lack of real-world SSB taxes at the time.^10^ There is now a critical need for the synthesis of literature on the outcomes of recently implemented SSB taxes to inform decision-making about the use of fiscal policy to create incentives for improving diet and health.

This study offers a systematic review and meta-analysis of the literature on implemented SSB taxes to provide comprehensive guidance on the outcomes associated with SSB taxation worldwide. It is part of a broader systematic review on the outcomes of fiscal and pricing policies on foods and nonalcoholic beverages commissioned by the World Health Organization (WHO). The review is intended to inform guidelines that will support WHO Member States in developing and implementing fiscal and pricing policies to promote healthy diets. This review is also expected to be of interest to policy makers in subnational jurisdictions and expand our understanding of effective policy approaches to improving public health.

## Methods

### Search Strategy

This systematic review and meta-analysis (CRD42019139426) adhered to the Preferred Reporting Items for Systematic Reviews and Meta-analyses (PRISMA) reporting guidelines^18^ and included peer-reviewed and grey literature from all countries and published in all languages from database inception through June 1, 2020. The review was guided by the Population, Intervention, Comparison and Outcome framework set by the WHO Nutrition Guidance Expert Advisory Group (NUGAG) Subgroup on Policy Actions, including *critical outcomes*, defined as price changes, taxed and untaxed beverage sales (including both store volume sold and household purchases), consumption (taxed SSBs and untaxed substitute beverages), and diet. Outcomes deemed by the NUGAG experts as *important* included product change (eg, reformulation), unintended consequences (eg, jobs, cross-border shopping), body weight status, diet-related NCDs, undernutrition, and pregnancy outcomes.

Peer-reviewed literature searches were performed in 8 bibliographic electronic databases, including Business Source Complete, Cochrane Central Register of Controlled Trials, Cochrane Database of Systematic Reviews, CINAHL Literature Plus with Full Text, EconLit, PsycINFO, PubMed, and Scopus. Fourteen sources of grey literature were used: Directory of Open Access Journals, EconPapers, EPPI-Centre Database of Promoting Health Effectiveness Reviews, EPPI-Centre Trials Register of Promoting Health Interventions, Google Scholar, HealthEvidence.org, Health Services Research Projects in Process, National Bureau of Economic Research, PDQ-Evidence for Informed Health Policymaking, ProQuest Dissertations and Theses Database, Social Science Research Network eLibrary, WHO Global Index Medicus, WHO International Clinical Trials Registry Platform, and WorldWideScience. Websites of relevant agencies and references from systematic reviews and papers selected for data extraction were checked. A University of Connecticut librarian assisted in developing the search strategy, which is presented with search results in eAppendix 1 in the Supplement.

### Eligibility Criteria

The review assessed population-level current or past fiscal (eg, taxes) and pricing policies (eg, minimum prices) on SSBs. In this context, SSBs refer to a broad set of nonalcoholic sugar-sweetened beverages (ie, beverages with added free sugars), which varied across policies and studies. Additionally, some SSB taxes were also applied to beverages sweetened with noncaloric sweeteners. The SSB tax type and size also varied across policies. Tax implementation was compared to not implementing a tax. We hypothesized that SSB taxes are associated with higher prices of taxed beverages, lower SSB sales and consumption, higher sales and consumption of untaxed beverages, and no changes in employment.

The review assessed the general population of children and adults (ages ≥18 years) across all countries and settings. Only primary studies or reports were considered, excluding opinion editorials, commentaries and reviews, modeling or simulation studies, and laboratory-based studies. Studies were included if they used one of the following research designs: randomized trials, interrupted time series designs, controlled and uncontrolled before and after studies, quasi-experimental designs, cross-sectional analyses using propensity score matching, difference-in-differences methods and fixed-effect analysis, longitudinal analyses using fixed effects, and ecological analysis. Studies were excluded if they did not include outcomes identified by the NUGAG committee.

### Data Collection and Extraction

At least 2 reviewers (T.A., K.M., and S.M.) independently screened titles and abstracts, assessed the full text of eligible articles, completed data extraction, and evaluated study quality. Any disagreement was resolved through consensus and discussion with another author (L.M.P.).

#### Quality of Study Assessment

As all studies were nonexperimental, their quality was assessed using a new tool adapted from a prior systematic review and meta-analysis of sugary drink taxes^15^ and informed by the Cochrane ROBINS-I risk of bias tool for nonrandomized studies of interventions.^19^ A new study quality tool (eTable 1 in the Supplement) was developed to capture multiple components of SSB tax evaluations focusing on the study design, validity of measures, sample representativeness and size, and adequate control for confounders. Assessment was done at the outcome rather than study level, as some papers included multiple study designs and data sets across outcomes in their analysis. Using 7 questions to assess the methodological rigor and data limitations, we assigned a score of low, medium, or high quality to each outcome in every reviewed paper.

#### Effect Size Extraction

For each article, 1 main effect size per outcome was selected, except when a study assessed more than 1 policy or used multiple data sets per outcome. Estimated changes across the entire posttax period were selected when available; alternatively, we used the latest reported posttax period. Where possible, estimated relative changes were extracted; when only absolute changes were reported, they were converted into relative changes by dividing both the estimated change and confidence intervals by baseline estimates. Volumetric measures were selected over measures of frequency or expenditure.

Where results of multiple models were presented, results were selected from the study authors’ preferred model; otherwise, the most fully controlled models were chosen. For substitution, the reported results were extracted for untaxed beverages or bottled water. If a study only provided estimates stratified by store, the store-level estimates were extracted and a meta-analysis was conducted to obtain a single estimate and confidence interval from the stratified estimates.

#### Missing Data

When uncertainty estimates or baseline data were not provided, study authors were contacted via email to request the missing data.

### Statistical Analysis

The synthesis of results proceeded in 2 stages. When a meta-analytic approach was feasible, results were meta-analyzed based on studies with complete data. Studies with missing data and those without statistical testing were analyzed narratively. For outcomes with few available studies or high heterogeneity across measures, a narrative synthesis of all studies was provided. In a narrative synthesis, results were aggregated by the direction of estimated results (eg, increase or decrease) and statistical significance of the estimates.

In addition to examining effect size estimates of outcomes for changes in demand for sales and consumption, measures of price elasticity and cross-price elasticity of demand were meta-analyzed. Price elasticity of demand is measured as percentage change in demand (sales or consumption) over percentage change in price, and cross-price elasticity is percentage change in demand for substitute products over percentage change in price for another product (SSBs in this review). eAppendix 2 in the Supplement provides details on the computation of the price elasticity measures.

Given that high heterogeneity of results was expected and that studies were nested within taxing jurisdictions, Hartung-Knapp adjusted 3-level random-effects models were used to generate pooled effect estimates using restricted maximum likelihood for estimating τ^2^ (eTable 4 in the Supplement).^20^ The proportion of variation in observed effect sizes that is due to variance in true effects, ie, heterogeneity, was assessed using the *I*^2^ statistic.^21^ In addition, 95% prediction intervals were estimated to provide a measure of the range of effect sizes expected from future studies, which accounts for both the variance in the estimated effect size and between-study heterogeneity (τ^2^).^22,23^

For the meta-analyzed outcomes, publication bias was assessed using the Egger test.^24^ Models were rerun excluding outliers and studies with the highest and lowest variance. Sensitivity analyses also included limiting the meta-analyses to high-quality studies. Meta-analyses were conducted in R version 4.1.0 (R Project for Statistical Computing),^25^ using the meta package version 4.19,^26^ with prediction intervals calculated using the metafor package.^27^ Auxiliary functions from the dmetar package (version 0.09.000) were used.^28^

## Results

### Study Selection and Characteristics

**Figure 1.**
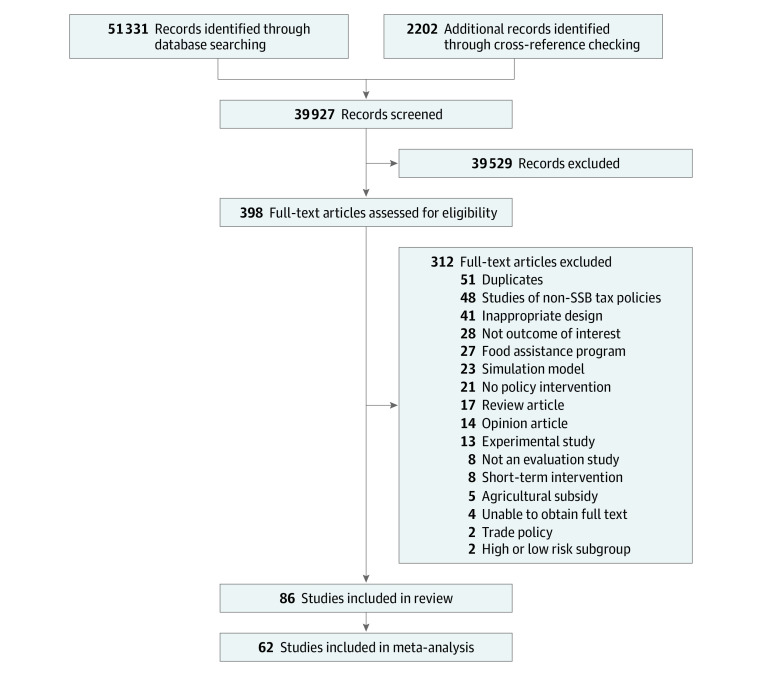
Study Flowchart SSB indicates sugar-sweetened beverage.

The search retrieved 39 927 unique titles for abstract and title screening, with 398 titles selected for full-text screening (Figure 1). We identified 86 articles^29,30,31,32,33,34,35,36,37,38,39,40,41,42,43,44,45,46,47,48,49,50,51,52,53,54,55,56,57,58,59,60,61,62,63,64,65,66,67,68,69,70,71,72,73,74,75,76,77,78,79,80,81,82,83,84,85,86,87,88,89,90,91,92,93,94,95,96,97,98,99,100,101,102,103,104,105,106,107,108,109,110,111,112,113,114^ that met all inclusion criteria: 61 peer-reviewed articles and 25 reports, dissertations, or working papers. No studies on pricing policies were identified.

### Location, Setting, and Study Characteristics

Most studies assessed 1 tax policy for multiple outcomes (eTable 2 in the Supplement). Most studies (n = 44) were evaluations of national taxes, including 17 studies for Mexico,^29,30,31,32,33,34,35,36,37,38,39,40,41,42,43,44,45^ 7 for the UK,^46,47,48,49,50,51,52^ 4 for France,^53,54,55,56^ 3 for Chile,^57,58,59^ 3 for Denmark,^56,60,61^ 2 for Barbados,^62,63^ 2 for Portugal,^64,65^ 1 for Finland,^56^ 1 for Hungary,^56^ 1 for Saudi Arabia,^66^ and 1 for South Africa.^67^ There were 42 articles evaluating local, state-level, or regional SSB taxes, including 13 studies for Philadelphia, Pennsylvania,^68,69,70,71,72,73,74,75,76,77,78,79,80^ 11 studies for Berkeley, California,^81,82,83,84,85,86,87,88,89,90,91^ 8 studies for state-level taxes in the United States,^88,92,93,94,95,96,97,98^ 4 studies for Oakland, California,^99,100,101,102^ 3 studies for Cook County, Illinois,^103,104,105^ 3 studies for Seattle, Washington,^106,107,108^ 3 studies for Catalonia, Spain,^109,110,111^ 1 study for San Francisco, California,^100^ 1 for Boulder, Colorado,^112^ 1 for Sheffield, United Kingdom,^113^ and 1 study for a UK restaurant chain.^114^

Most studies provided evidence for prices (n = 49),^29,31,32,36,38,41,42,46,47,53,54,55,56,57,58,59,60,61,62,64,65,66,67,68,72,73,77,79,80,81,82,83,86,87,88,89,91,93,99,100,101,103,105,106,107,108,109,111,112^ followed by SSB sales (n = 43),^29,30,33,34,35,37,39,40,41,42,44,52,54,56,57,59,61,63,64,65,66,69,70,71,72,73,76,79,82,86,87,88,89,90,92,93,99,104,106,109,111,113,114^ sales of substitution beverages (n = 33),^29,33,34,35,37,39,40,42,44,52,54,56,57,59,61,63,66,69,70,71,72,73,76,88,89,90,99,104,106,109,111,113,114^ unintended consequences (n = 15),^43,50,51,70,71,72,73,76,78,79,89,99,102,104,106^ SSB consumption (n = 13),^45,70,74,75,84,85,89,94,95,97,99,107,110^ and consumption of substitution beverages (n = 11).^70,74,75,84,85,89,94,95,99,107,110^ Few studies assessed product changes (n = 6),^47,48,49,52,65,67^ body mass index (BMI; n = 5),^94,95,96,97,98^ and dietary intake (n = 2).^94,95^ No studies were identified on pregnancy, undernutrition, and diet-related NCDs. All studies used nonexperimental research designs.

### Study Quality

The quality of studies was highly variable (eTable 2 in the Supplement). Studies measuring consumption (SSB or substitution) were generally of low quality (10 of 13 [77%] for SSB consumption; 9 of 11 [82%] for consumption of substitutes), while the majority of price and sales evaluations were rated as high quality. The available BMI and diet evaluations were deemed as medium quality.

### Synthesis of Results

Sixty-two articles^29,30,31,32,33,34,35,36,37,38,39,40,46,47,53,54,55,57,58,59,60,61,62,63,64,66,67,68,69,70,72,73,74,75,80,81,82,83,84,85,86,87,88,89,90,91,92,99,100,101,103,104,105,106,107,108,109,110,111,112,113,114^ (72%) were included in at least 1 of the 7 meta-analyses conducted: (1) change in prices (tax pass-through), (2) percentage change in demand measured by SSB sales, (3) SSB sales (price elasticity), (4) sales of substitute products (cross-price elasticity), (5) percentage change in demand and/or SSB consumption, (6) SSB consumption (price elasticity), and (7) consumption of substitute products (cross-price elasticity). Results from the remaining 24 articles^41,42,43,44,45,48,49,50,51,52,56,65,71,76,77,78,79,93,94,95,96,97,98,102^ were synthesized narratively. For the meta-analyzed outcomes, 15 studies^41,42,44,45,52,56,65,71,76,77,79,93,94,95,97^ were excluded from the meta-analysis because of missing data. A narrative synthesis was conducted for BMI, diet quality, product change, and unintended consequences.

### Results From Meta-analyses

**Figure 2.**
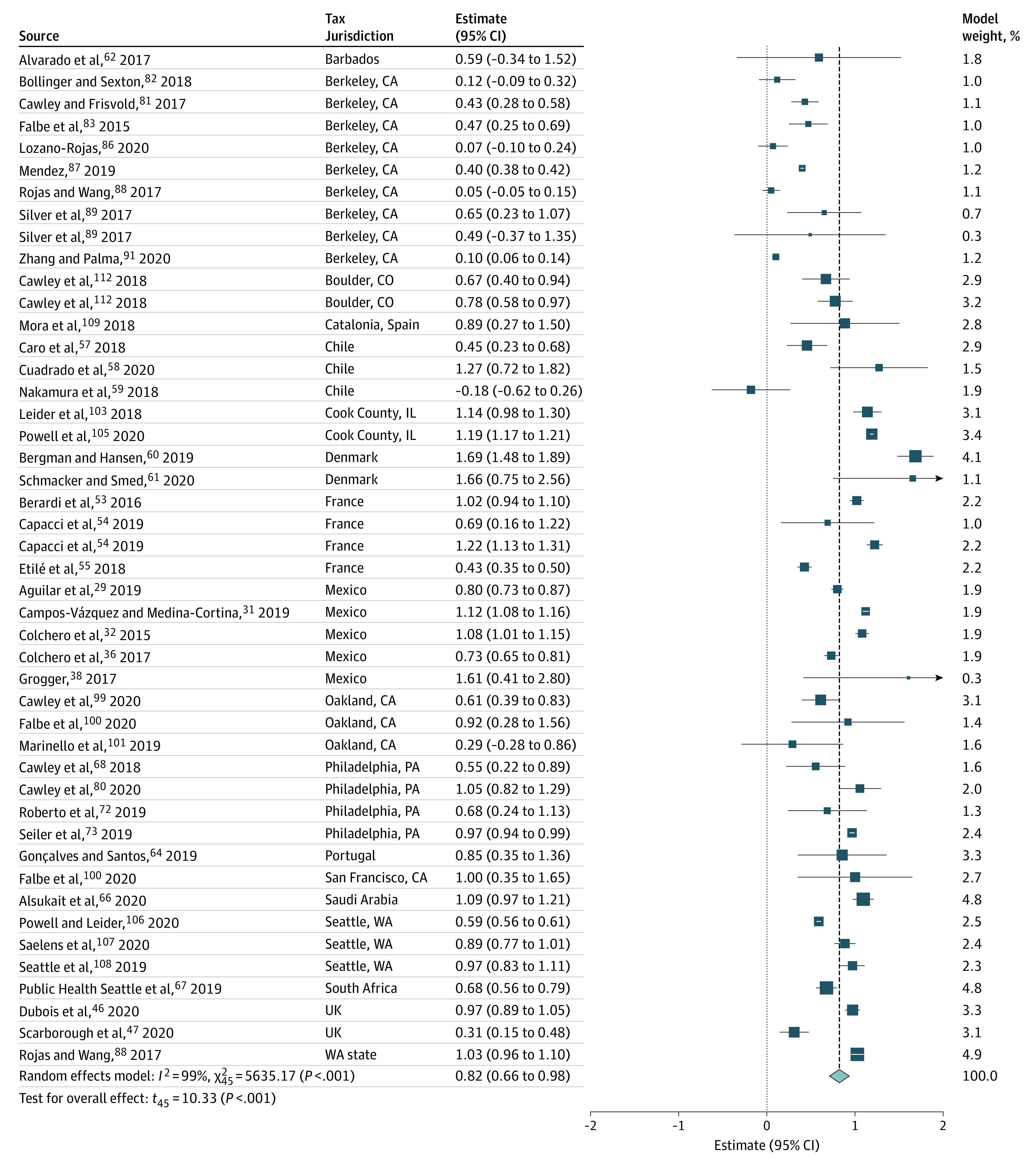
Meta-analysis of Price Outcomes Following Sugar-Sweetened Beverage Taxes: Tax Pass-Through

Summary results from all meta-analyses are presented in Table 1. Price outcomes had the largest body of evidence, with 46 estimates from 41 articles^29,31,32,36,38,46,47,53,54,55,57,58,59,60,61,62,64,66,67,68,72,73,80,81,82,83,86,87,88,89,91,99,100,101,103,105,106,107,108,109,112^ for 18 tax policies. There was evidence of a significant increase in prices of taxed beverages and high heterogeneity. Overall tax pass-through (the extent to which taxes were passed on to consumers in the form of higher prices) of the evaluated SSB taxes was estimated at 82% (95% CI, 66%-98%; *P* < .001; prediction interval, 9%-156%; *I*^2^ = 99.2%; 95% CI, 99.1%-99.3%; *P* < .001) (Figure 2). That is, a 10%-equivalent SSB tax was estimated to increase consumer prices of taxed beverages by 8.2%, suggesting an incomplete pass-through and tax undershifting.

**Table 1.** Meta-analysis of Outcomes Following SSB Taxes

Outcome	No.			3-Level random-effects model						
	Estimates	Articles	Tax policies	Pooled estimate (95% CI)	*P* value	Prediction interval	Q for heterogeneity	*P* value	Heterogeneity I^2^ (95% CI),	Publication bias
Price: tax pass-through, %	46	41	18	82.2 (66.2 to 98.3)	<.001	8.6 to 155.9	5635	<.001	99.2 (99.1 to 99.3)	None
SSB sales: % demand change	35	33	16	−14.6 (−20.4 to −8.8)	<.001	−37.6 to 8.4	709 742	<.001	100 (NA)	None
SSB sales: price elasticity	35	33	16	−1.59 (−2.11 to −1.08)	<.001	−3.94 to 0.75	122 929	<.001	100 (NA)	None
Sales, substitution beverages: cross-price elasticity	25	24	14	0.42 (−0.52 to 1.35)	.37	−3.69 to 4.52	1056	<.001	97.7 (97.3 to 98.1)	Yes
SSB consumption: % demand change	12	9	5	−18.1 (−37.6 to 1.5)	.07	−60.8 to 24.6	23	.02	52.9 (9.4 to 75.6)	Yes
SSB consumption: price elasticity	12	9	5	−3.78 (−8.86 to 1.30)	.13	−15.78 to 8.22	60	<.001	81.6 (68.9 to 89.1)	None
Consumptionof substitution beverages: cross-price elasticity	12	9	5	0.54 (−0.60 to 1.68)	.32	−1.70 to 2.79	21	.03	47.6 (0 to 73.1)	None

Abbreviations: NA, not applicable; SSB, sugar-sweetened beverages.

**Figure 3.**
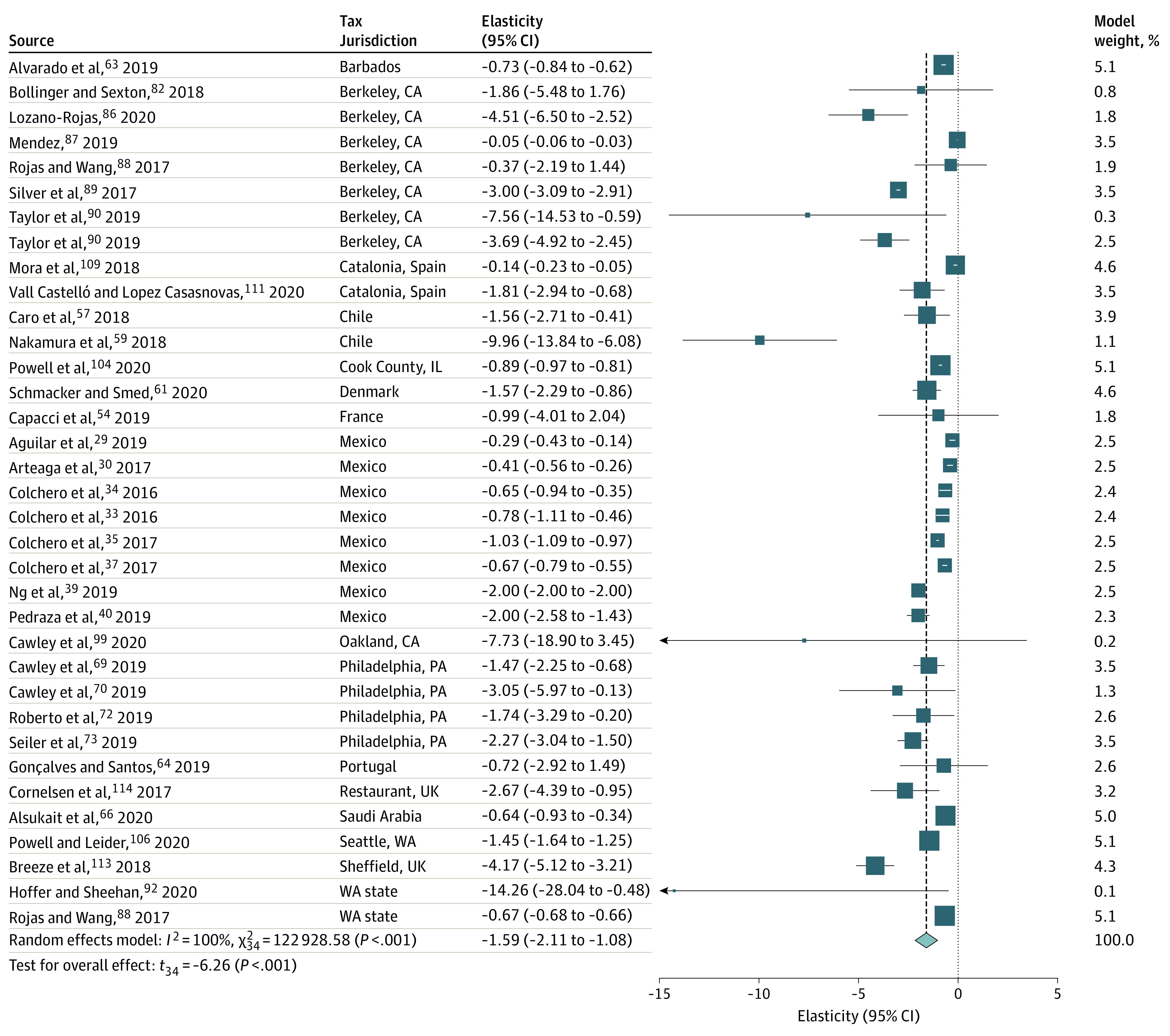
Meta-analysis of Sugar-Sweetened Beverage Sales Following Sugar-Sweetened Beverage Taxes: Price Elasticity of Demand for Taxed Beverages

Meta-analyzed results for SSB sales were based on 35 estimates from 33 studies^29,30,33,34,35,37,39,40,54,57,59,61,63,64,66,69,70,72,73,82,86,87,88,89,90,92,99,104,106,109,111,113,114^ for 16 tax policies. The meta-analyzed estimate for price elasticity for SSB sales was −1.59 (95% CI, −2.11 to −1.08; *P* < .001; prediction interval, −3.94 to 0.75; *I*^2^ = 100%) (Figure 3). Across all studies and tax policies, there was a significant reduction in SSB sales of 15% (95% CI, −20% to −9%; *P* < .001; prediction interval, −38% to 8%; *I*^2^ = 100%) (eFigure 1 in the Supplement). There was no evidence of significant substitution to sales of untaxed beverages (eFigure 2 in the Supplement).

The meta-analyzed estimates for SSB demand measured by consumption were not statistically significant. Consumption of taxed beverages in 9 studies^70,74,75,84,85,89,99,107,110^ (12 estimates) for 5 tax policies was estimated to have a price elasticity of −3.78 (95% CI, −8.86 to 1.30; *P* = .13) (eFigure 3 in the Supplement) and an estimated decline in demand of 18% (95% CI: −38 to 1%; *P* = .07) (eFigure 4 in the Supplement). Additionally, there was no significant change in the consumption of untaxed beverages in 9 studies^70,74,75,84,85,89,99,107,110^ (eFigure 5 in the Supplement).

### Sensitivity and Subgroup Analysis

Results of the overall meta-analyses were consistent across several sensitivity checks, including removal of outlier studies and limiting the analyses to high-quality studies (eTable 3 in the Supplement). In no cases did removal of outliers or subanalysis of high-quality studies lead to a substantive change in the magnitude or statistical significance of the pooled results. Heterogeneity remained substantial even after outlier studies were removed (*I*^2^ > 75%). For example, removing 13 outliers from the price meta-analysis did not change the estimated result (pass-through of 84% vs overall 82%; *P* < .001), but reduced heterogeneity (Q from 5635 to 225; *I*^2^ from 99% to 86%). The subset of studies ranked as high quality (n = 32) was estimated to have an almost identical pass-through of 79% (*P* < .001). Consistent results also were seen for the price elasticity of demand based on SSB sales: −1.57 (*P* < .001) when excluding outliers and −1.39 (*P* < .001) in the subgroup of high quality studies.

### Publication Bias

There was no evidence of publication bias for studies assessing SSB prices and sales. Publication bias was detected by the Egger test for sales of substitution beverages and SSB consumption; their funnel plots are presented in eFigure 6 in the Supplement.

### Narrative Synthesis

Studies with missing data for the meta-analyzed outcomes suggested similar results, including higher prices of taxed beverages and reduced sales (Table 2).

**Table 2.** Summary of Narrative Synthesis Results for SSB Tax Outcomes

Outcome	No. of studies	Tax policy	Tax jurisdiction/location	Direction and statistical significance of estimated outcome(s)	Primary measures
**Meta-analyzed outcomes from studies in narrative analysis only**					
Prices: tax pass-through	5	Single-tier volume-based excise tax	Denmark, Finland, France, Hungary, Mexico, and Philadelphia, Pennsylvania	Increase, no statistical testing (Andalón and Gibson,^41^ 2017; Bonilla-Chacin et al,^42^ 2016; ECSIPC,^56^ 2014; Coary and Baskin,^77^ 2018; Oxford Economics,^79^ 2017)	Price change of taxed beverages
	2	Tiered volume-based excise tax	Portugal and Catalonia, Spain	Increase, no statistical testing (Goiana-da-Silva et al,^65^ 2020; Vall Castelló and Lopez Casasnovas,^111^ 2020)	Price change of taxed beverages
	1	Sales tax	United States	No significant change (Colantuoni and Rojas,^93^ 2015). Increase, significant (Colantuoni and Rojas,^93^ 2015)	Price change of taxed beverages (soft drinks)
Sales of taxed beverages	7	Single-tier volume-based excise tax	Denmark, Finland, France, Hungary, Mexico, and Philadelphia, Pennsylvania	Decrease, no statistical testing (Andalón and Gibson,^41^ 2017; Bonilla-Chacin et al,^42^ 2016; ECSIPC,^56^ 2014; Pizzutti,^71^ 2019; Oxford Economics^79^ 2017). Decrease, significant (Pedraza et al,^44^ 2018; Baskin and Coary,^76^ 2019)	Change in volume sold of taxed beverages, change in sales for taxed beverages
	2	Tiered volume-based excise tax	Portugal and United Kingdom	Decrease, significant (Goiana-da-Silva et al,^65^ 2020). Decrease, no statistical testing (Public Health England,^52^ 2019)	Change in volume sold of taxed beverages
	1	Sales tax	United States	No significant change (Colantuoni and Rojas,^93^ 2015)	Change in volume sold of soft drinks
Sales of substitution beverages	7	Single-tier volume-based excise tax	Denmark; Mexico; Saudi Arabia; Berkeley, California; Philadelphia, Pennsylvania	No significant change (Aguilar et al,^29^ 2019; Baskin and Coary,^76^ 2019). No change, no statistical testing (ECSIPC,^56^ 2014; Alsukait et al,^66^ 2020). Increase, significant (Taylor et al,^90^ 2019). Increase, no statistical testing (Pizzutti,^71^ 2019). Mixed results (Pedraza et al,^44^ 2018)	Change in volume sold of untaxed beverages, change in sales for untaxed beverages
	1	Tiered volume-based excise tax	United Kingdom	Increase, no statistical testing (Public Health England,^52^ 2019)	Change in volume sold of taxed beverages
Consumption of taxed beverages	3	Sales tax	United States	No significant change (Fletcher et al,^94^ 2015; Fletcher et al,^97^ 2010). Decrease, significant (Fletcher et al,^95^ 2010)	Change in volume consumed (soft drinks)
	1	Single-tier volume-based excise tax	Mexico	Decrease, significant (Sánchez-Romero et al,^45^ 2020)	Probability of consumption levels
Consumption, substitution beverages	2	Sales tax	United States	Increase, significant (Fletcher et al,^94^ 2015). Mixed results (Fletcher et al,^95^ 2010)	Change in intake of untaxed beverages
**Outcomes not included in meta-analyses, narrative synthesis only**					
Cross-border shopping	7	Single-tier volume-based excise tax	Cook County, Illinois; Oakland, California; Philadelphia, Pennsylvania; and Seattle, Washington	Increase (Cawley et al,^70^ 2019; Seiler et al,^73^ 2019; Powell et al,^104^ 2020). Increase, no statistical testing (Pizzutti,^71^ 2019; Oxford Economics,^79^ 2017). Mixed results (Cawley et al,^99^ 2020). No significant change (Powell and Leider,^106^ 2020)	Increased taxed beverage sales in nearby tax-free areas
Retailer sales revenue	4	Single-tier volume-based excise tax	Berkeley, California, and Philadelphia, Pennsylvania	Decrease, significant (Baskin and Coary,^76^ 2019). Mixed results (Roberto et al,^72^ 2019). Increase, no statistical testing (Silver et al,^89^ 2017). Decrease, no statistical testing Oxford Economics,^79^ 2017)	Reduced total sales in taxed jurisdictions
Employment	2	Single-tier volume-based excise tax	Mexico and Philadelphia, Pennsylvania	No significant change (Guerrero-Lopez et al,^43^ 2017; Lawman et al,^78^ 2019). Decrease, significant (Guerrero-Lopez et al,^43^ 2017)	Unemployment; employment in beverage manufacturing
Other	2	Tiered volume-based excise tax	United Kingdom	No significant change (Law et al,^50^ 2020; Law et al,^51^ 2020)	Turnover (soft drink manufacturing), market return
	1	Single-tier volume-based excise tax	Oakland, California	No significant change (Zenk et al,^102^ 2020)	Store advertising
Product change or reformulation	6	Tiered volume-based excise tax	Portugal and United Kingdom	Decrease, no statistical testing (Chu et al,^48^ 2020; Hashem et al,^49^ 2019; Public Health England,^52^ 2019; Goiana-da-Silva,^65^ 2020). Decrease, significant (Scarborough et al,^47^ 2020)	Sugar content, beverage energy content and density
		Tiered sugar-based excise tax	South Africa	Decrease, no statistical testing (Stacey et al,^67^ 2019)	
Body weight	5	Sales tax	United States	No significant change (Fletcher et al,^94^ 2015; Fletcher et al,^95^ 2010; Fletcher et al,^97^ 2010; Pak,^98^ 2013). Decrease, significant (Fletcher et al,^96^ 2010)	Body mass index, overweight, obesity
Dietary intake/quality	2	Sales tax	United States	No significant change (Fletcher et al,^95^ 2010). Increase, significant (Fletcher et al,^94^ 2015)	Nutrient intake, total calories

Abbreviations: ECSIPC, European Competitiveness and Sustainable Industrial Policy Consortium; SSB, sugar-sweetened beverages.

Unintended consequences had studies in several areas: cross-border shopping (ie, increased sales of taxed beverages in areas adjacent to taxing jurisdictions); retailer revenue; employment and unemployment; and other factors (market return, turnover for beverage manufacturers, exterior and interior store advertising for SSBs). For local US taxes, most studies on cross-border shopping pointed to a significant increase^70,73,104^ or an increase that was not statistically tested.^71,79^ However, there were studies with statistically significant findings only for certain measures of cross-border shopping^99^ or none at all.^106^ Several studies also showed a reduction in total grocery sales for all^76^ or some retailers.^72^ Evaluations of national taxes did not assess cross-border shopping or retailer revenue outcomes. Unemployment changes due to SSB taxes were identified as null in a Philadelphia-based study^78^ and a Mexico-based study found no change in manufacturing jobs and lower national unemployment rates.^43^ There were no significant posttax changes for the other factors, including store SSB advertising and price promotions,^102^ market return,^51^ and turnover for UK soft drink manufacturers.^50^

BMI outcomes were assessed for US-based sales taxes only, with no association identified in 4 studies^94,95,97,98^ and a negative association in 1 study.^96^ Similarly, diet changes were assessed for small US sales taxes, with no change in total calorie intake in 1 study^95^ and increased intake in another.^94^ No evidence was available yet for BMI and dietary outcomes based on recent excise taxes in either the US or globally.

All 6 studies^47,48,49,52,65,67^ on product changes in the case of tiered taxes found evidence of beverage reformulation and reduction in sugar content. One study^47^ provided statistical testing to show beverage reformulation following the UK Soft Drinks Industry Levy and found a significant reduction in the share of beverages exceeding the lower levy threshold for sugar.

### Results From Subpopulation Analyses

Only a fraction of studies included subpopulation comparisons, particularly for outcomes other than sales. Evaluations of US-based local taxes had very limited data across population groups. We completed a subgroup analysis by income or socioeconomic status (SES) only; comparisons by other sociodemographic characteristics were rare. The definition of SES varied across studies.

Overall, results on subpopulation differences were mixed across countries. Not all studies formally tested group differences. For sales, the evidence from Mexico was consistent in identifying higher reductions in SSB sales for low-income or low-SES households.^34,35,37,39,42,44^ Findings in other countries were less consistent. For example, 1 Philadelphia-based study showed no difference in SSB sales by income, race, or ethnicity,^70^ while another study from this city found a lower reduction in SSB sales in low-income residential areas.^73^ Four studies reported greater declines in SSB sales for higher income groups or areas, including in Chile^57,59^ and Catalonia, Spain.^109,111^ A UK-based evaluation showed that the reduction in sugar purchased per household from taxed beverages was the smallest in the lowest SES group (9% vs 24% overall).^52^

Inconsistent findings were observed in the data on subgroup differences for beverage substitution. There was little by-group data on consumption of SSB substitutes, including findings of no variation by income in the consumption results in Philadelphia^70^ and Mexico.^45^ The Philadelphia study identified heterogeneity in posttax SSB consumption across other sociodemographic characteristics, including larger effect sizes and a statistically significant reduction for African American children.^70^ Finally, 1 study on BMI and SSB taxes reported larger changes among female individuals, middle-aged and older individuals, and individuals with greater education, with varied findings across racial and ethnic groups.^96^

## Discussion

We have conducted a comprehensive systematic review and meta-analysis of worldwide published and grey literature on the outcomes associated with implemented fiscal and pricing policies on SSBs. The evidence suggests several important conclusions about the outcomes following implementation of SSB taxes and implications for improving nutrition and health.

Most SSB tax evaluations focused on posttax changes in prices and sales. There is conclusive evidence that SSB taxes are associated with higher prices of taxed beverages and lower sales, suggesting that consumers respond to economic interventions. Across all studies and SSB tax policies worldwide, we found an 82% tax pass-through rate and highly sensitive demand for SSBs, with an estimated price elasticity of −1.59 for SSB sales. Given that many SSB taxes to date have been relatively small (ie, raising prices by ≤10%) with an incomplete pass-through, the average reduction in sales of taxed beverages was approximately 15%. The findings for prices and sales come from overwhelmingly high-quality studies, and the findings from the meta-analysis were robust to multiple sensitivity analyses. Studies of beverage sales found no evidence, on average, of substitution to untaxed beverages.

Whereas study quality was generally high for price and sales evaluations, consumption assessments were often deemed as low quality. Large representative studies to identify changes in SSB consumption for both children and adults are currently lacking. Meta-analyzed estimates of tax-related changes in consumption were not statistically significant, potentially due to a small number of studies with limited statistical power. Just as for sales, there was no evidence of substitution toward untaxed beverages based on consumption studies.

The data are currently not granular enough to enable analyses of tax outcomes for population subgroups. Most studies provide aggregate results for the general population, with only a small subset of research reporting data for subpopulations, usually by SES or household income. This is likely due to the frequent reliance of tax evaluations on retailer-based scanner data aggregated at the store level. Some national tax evaluations have used household consumer panels where income and limited sociodemographic variables are available. As only a fraction of studies included subgroup analyses, it is unlikely that income and/or SES differences account for much of the heterogeneity in the overall results. Future research should focus on understanding heterogeneity of policy response across subpopulations, including racial and ethnic differences and the equity impacts of SSB taxes.

Tiered taxes were associated with beverage reformulation and reduced sugar content of taxed beverages. Unintended consequences were detected only in the case of local SSB taxes in the United States, where, in some cases, there was evidence of cross-border shopping and reduced revenue among local retailers. Literature on employment and SSB taxes is still limited, but so far there is no evidence of a negative association between SSB taxes and jobs. Longer-term studies are needed to assess how changes in SSB taxes are associated with dietary intake, BMI, and health outcomes. Prior studies on BMI and SSB taxes were limited to research on low state sales taxes, which are unlikely to adequately represent potential changes in BMI outcomes of recent excise SSB taxes. Most SSB taxes are recent phenomena, and not enough time has passed to allow for such evaluations. Research on the long-term outcomes of implemented excise SSB taxes will be necessary. It is also important to acknowledge that the effectiveness of SSB taxes could change over time, and future research should compare immediate vs longer-term outcomes.

Results from this review align with evidence on the outcomes of fiscal policies to reduce consumption of other so-called sin products, including tobacco and alcohol. Governments around the world have increasingly used excise taxes on these products to discourage consumption and reduce adverse health consequences, with documented success.^115^ Additionally, similar to our results on employment, tobacco and alcohol taxes were shown to have no negative overall impact on employment.^115^

### Limitations

This study has limitations. Multiple outcomes could not be meta-analyzed due to a low number of available studies. The selection of outcomes was predetermined by the NUGAG committee, and therefore, the review did not include outcomes of potential interest, such as tax revenue. For several key outcomes, particularly SSB prices and sales, the heterogeneity was very high, likely reflecting the variation in the study design, quality, and data sources. We have attempted to account for the variation in the tax designs by estimating 3-level random effects models with tax jurisdiction as 1 level (clustering). In our assessment of heterogeneity caused by tax jurisdiction and between-study variation (eTable 4 in the Supplement), we found that outcomes vary in the magnitude of heterogeneity contributed by tax jurisdiction (eg, price elasticity for consumption shows large heterogeneity [high τ^2^] associated with tax jurisdiction). This is likely caused by the large differences in effect sizes seen between studies from different regions (eFigure 3 in the Supplement). The high *I*^2^ values identified in this study suggest that most of the variability across studies is because of heterogeneity rather than sampling error.^21^ More country- and jurisdiction-specific studies are needed to capture regional variability in effects to provide a fuller picture of the outcomes of SSB taxation across the globe. Additionally, stratifying by type of store or type of study design was not feasible given the low number of studies in each subgroup.

## Conclusions

This systematic review and meta-analysis of implemented SSB taxes worldwide found evidence that consumers respond to economic interventions; the review showed that SSB taxes were associated with higher prices of taxed beverages and lower sales. Further research on SSB taxes is needed to understand associations with diet and health outcomes and to assess heterogeneity of consumer responses to improve policy reach and effectiveness.

## Notes

Supplement. **eAppendix 1. **Search Terms and Search Results **eAppendix 2.** Computation of Price Elasticity of Demand and Cross-Price Elasticity of Demand Measures **eTable 1.** Quality Assessment of Fiscal Policy Studies **eTable 2.** Characteristics of Studies **eTable 3.** Summary of Sensitivity and Sub-group Meta-Analyses **eTable 4.** Sources of Heterogeneity (τ^2^) for 3-Level Random-Effects Models **eFigure 1.** Meta-analysis of SSB Sales Following SSB Taxes: Percentage Change in Demand for Taxed Beverages **eFigure 2.** Meta-analysis of Substitution Beverage Sales Following SSB Taxes: Cross-Price Elasticity **eFigure 3.** Meta-analysis of SSB Consumption Following SSB Taxes: Price Elasticity of Demand for Taxed Beverages **eFigure 4.** Meta-analysis of SSB Consumption Following SSB Taxes: Percentage Change in Demand for Taxed Beverages **eFigure 5.** Meta-analysis of Consumption of Substitution Beverages Following SSB Taxes: Cross-Price Elasticity **eFigure 6.** Publication Bias: Funnel Plots
