# Supplementary material for: Outcomes Following Taxation of Sugar-Sweetened Beverages: A Systematic Review and Meta-analysis
Source: JAMA Netw Open. 2022 Jun 1;5(6):e2215276. doi: 10.1001/jamanetworkopen.2022.15276 (PMC9161017; doi:10.1001/jamanetworkopen.2022.15276)
Supplement: Supplement. — eAppendix 1. Search Terms and Search Results eAppendix 2. Computation of Price Elasticity of Demand and Cross-Price Elasticity of Demand Measures eTable 1. Quality Assessment of Fiscal Policy Studies eTable 2. Characteristics of Studies eTable 3. Summary of Sensitivity and Sub-group Meta-Analyses eTable 4. Sources of Heterogeneity (τ2) for 3-Level Random-Effects Models eFigure 1. Meta-analysis of SSB Sales Following SSB Taxes: Percentage Change in Demand for Taxed Beverages eFigure 2. Meta-analysis of Substitution Beverage Sales Following SSB Taxes: Cross-Price Elasticity eFigure 3. Meta-analysis of SSB Consumption Following SSB Taxes: Price Elasticity of Demand for Taxed Beverages eFigure 4. Meta-analysis of SSB Consumption Following SSB Taxes: Percentage Change in Demand for Taxed Beverages eFigure 5. Meta-analysis of Consumption of Substitution Beverages Following SSB Taxes: Cross-Price Elasticity eFigure 6. Publication Bias: Funnel Plots [file jamanetwopen-e2215276-s001.pdf]

Supplemental Online Content

Andreyeva T, Marple K, Marinello S, Moore TE, Powell LM. Outcomes following taxation of sugar-sweetened beverages: a systematic review and meta-analysis. *JAMA Netw Open.* 2022;5(6):e2215276. doi:10.1001/jamanetworkopen.2022.15276

eAppendix 1. Search Terms and Search Results

eAppendix 2. Computation of Price Elasticity of Demand and Cross-Price Elasticity of Demand Measures

eTable 1. Quality Assessment of Fiscal Policy Studies

eTable 2. Characteristics of Studies

eTable 3. Summary of Sensitivity and Sub-group Meta-Analyses

eTable 4. Sources of Heterogeneity (τ^2) for 3-Level Random Effects Models

eFigure 1. Meta-analysis of SSB Sales Following SSB Taxes: Percentage Change in Demand for Taxed Beverages

eFigure 2. Meta-analysis of Substitution Beverage Sales Following SSB Taxes: Cross-Price Elasticity

eFigure 3. Meta-analysis of SSB Consumption Following SSB Taxes: Price Elasticity of Demand for Taxed Beverages

eFigure 4. Meta-analysis of SSB Consumption Following SSB Taxes: Percentage Change in Demand for Taxed Beverages

eFigure 5. Meta-analysis of Consumption of Substitution Beverages Following SSB Taxes: Cross-Price Elasticity

eFigure 6. Publication Bias: Funnel Plots

This supplemental material has been provided by the authors to give readers additional information about their work.

© 2022 Andreyeva T et al. *JAMA Network Open.*

eAppendix 1. Search Terms and Search Results

PubMed Search Terms

Concept #1: TREATMENT OR INTERVENTION

“Taxes”[MeSH] OR “Fiscal Policy”[MeSH] OR “Health Policy/economics”[MeSH] OR “Healthy Diet/economics” [MeSH] OR “Health Promotion/economics”[MeSH] OR “Consumer Behavior/economics”[MeSH] OR “Diet/economics”[MeSH] OR “Carbonated Beverages/economics”[MeSH] OR “Government Programs/economics”[MeSH] OR “Public Health/economics”[MeSH] OR tax[tiab] OR taxes[tiab] OR taxation[tiab] OR taxed[tiab] OR taxing[tiab] OR subsidy[tiab] OR subsidies[tiab] OR subsidized[tiab] OR coupon[tiab] OR coupons[tiab] OR incentive[tiab] OR incentives[tiab] OR disincentive[tiab] OR disincentives[tiab] OR discount[tiab] OR discounts[tiab] OR discounted[tiab] OR pricing[tiab] OR priced[tiab] OR “price reduction”[tiab] OR “price increase”[tiab] OR “price promotion”[tiab] OR “price promotions”[tiab] OR “price floor”[tiab] OR “price ceiling”[tiab] OR “price cap”[tiab] OR “price caps”[tiab] OR “pricing policy”[tiab] OR “pricing policies”[tiab] OR “fiscal policy”[tiab] OR “fiscal policies”[tiab] OR voucher[tiab] OR vouchers[tiab] OR rebate[tiab] OR rebates[tiab] OR levy[tiab]

AND

Concept #2: TARGETS FOR TREATMENT OR INTERVENTION

"Food"[MeSH] OR "Food and Beverages"[MeSH] OR "Beverages"[MeSH] OR “Sugars”[MeSH] OR “Fruit”[MeSH] OR “Vegetables”[MeSH] OR “Healthy Food”[MeSH] OR "Carbonated Beverages"[MeSH] OR “Dairy Products”[MeSH] OR “Meat”[MeSH] OR “Dietary Fats”[MeSH] OR “Fishes”[MeSH] OR “Seafood”[MeSH] OR "Infant formula"[MeSH] OR
“sugary drink”[tiab] OR “sugary drinks”[tiab] OR “sugary beverages”[tiab] OR “sweetened beverage”[tiab] OR “sweetened beverages”[tiab] OR “sweetened drink”[tiab] OR “sweetened drinks”[tiab] OR “sugar-sweetened beverage”[tiab] OR “sugar-sweetened beverages”[tiab] OR “sugar-sweetened drink”[tiab] OR “sugar-sweetened drinks”[tiab] OR “carbonated beverage”[tiab] OR “carbonated beverages”[tiab] OR “carbonated-beverage”[tiab] OR “carbonated-beverages”[tiab] OR “carbonated drink”[tiab] OR “carbonated drinks”[tiab] OR “carbonated-drink”[tiab] OR “carbonated-drinks”[tiab] OR “high-calorie drink”[tiab] OR “high-calorie drinks”[tiab] OR “high calorie drink”[tiab] OR “high calorie drinks”[tiab] OR “high-calorie beverage”[tiab] OR “high-calorie beverages”[tiab] OR “high calorie beverage”[tiab] OR “high calorie beverages”[tiab] OR “low-calorie drink”[tiab] OR “low-calorie drinks”[tiab] OR “low calorie drink”[tiab] OR “low calorie drinks”[tiab] OR “low-calorie beverage”[tiab] OR “low-calorie beverages”[tiab] OR “low calorie beverage”[tiab] OR “low calorie beverages”[tiab] OR “soft drink”[tiab] OR “soft drinks”[tiab] OR “soft-drink”[tiab] OR “soft-drinks”[tiab] OR “fizzy drink”[tiab] OR “fizzy drinks”[tiab] OR “fizzy-drink”[tiab] OR “fizzy-drinks”[tiab] OR “energy drink”[tiab] OR “energy drinks”[tiab] OR “energy-drink”[tiab] OR “energy-drinks”[tiab] OR “fruit drink”[tiab] OR “fruit drinks”[tiab] OR “fruit-drink”[tiab] OR “fruit-drinks”[tiab] OR “sports

© 2022 Andreyeva T et al. *JAMA Network Open.*

drink”[tiab] OR “sports drinks”[tiab] OR “sports-drink”[tiab] OR “sports-drinks”[tiab] OR tea[tiab] OR teas[tiab] OR coffee[tiab] OR coffees[tiab] OR soda[tiab] OR sodas[tiab] OR cola[tiab] OR colas[tiab] OR “flavored water”[tiab] OR “vitamin water”[tiab] OR SSB*[tiab] OR “soda pop”[tiab] OR “chocolate milk”[tiab] OR “flavored milk”[tiab] OR juice[tiab] OR syrup*[tiab] OR

“healthy food”[tiab] OR “healthy foods”[tiab] OR “unhealthy food”[tiab] OR “unhealthy foods”[tiab] OR “nutritious foods”[tiab] OR “junk food”[tiab] OR “junk foods”[tiab] OR “energy dense food”[tiab] OR “high-calorie food”[tiab] OR “highcalorie food”[tiab] OR “high calorie food”[tiab] OR “energy dense foods”[tiab] OR “high-calorie foods”[tiab] OR “highcalorie foods”[tiab] OR “high calorie foods”[tiab] OR “low-calorie food”[tiab] OR “lowcalorie food”[tiab] OR “low calorie food”[tiab] OR “low-calorie foods”[tiab] OR “lowcalorie foods”[tiab] OR “low calorie foods”[tiab] OR "highenergy food"[tiab] OR "high energy food"[tiab] OR "high-energy food"[tiab] OR "highenergy foods"[tiab] OR "high energy foods"[tiab] OR "high-energy foods"[tiab] OR "lowenergy food"[tiab] OR "low energy food"[tiab] OR "low-energy food"[tiab] OR "lowenergy foods"[tiab] OR "low energy foods"[tiab] OR "low-energy foods"[tiab] OR “low-fat”[tiab] OR “low fat”[tiab] OR “lowfat”[tiab] OR

“fatty food”[tiab] OR “fatty foods”[tiab] OR “fattening food”[tiab] OR “fattening-food”[tiab] OR “salty food”[tiab] OR “salty-food”[tiab] OR “processed food”[tiab] OR “fattening foods”[tiab] OR “fattening-foods”[tiab] OR “salty foods”[tiab] OR “salty-foods”[tiab] OR “processed foods”[tiab] OR “takeout food”[tiab] OR “take-out food”[tiab] OR “takeaway food”[tiab] OR “take-away food”[tiab] OR “takeout foods”[tiab] OR “take-out foods”[tiab] OR “takeaway foods”[tiab] OR “take-away foods”[tiab] OR “fast food”[tiab] OR “fast-food”[tiab] OR “fast foods”[tiab] OR “fast-foods”[tiab] OR fastfood[tiab] OR “healthy diet"[tiab] OR "healthy diets”[tiab] OR "unhealthy diet"[tiab] OR "unhealthy diets"[tiab] OR

fruit[tiab] OR fruits[tiab] OR vegetable[tiab] OR vegetables[tiab] OR legumes[tiab] OR pulses[tiab] OR beans[tiab] OR lentils[tiab] OR peas[tiab] OR peanut*[tiab] OR salad[tiab] OR potato[tiab] OR potatoes[tiab] OR burger[tiab] OR candy[tiab] OR candies[tiab] OR confectionar*[tiab] OR chips[tiab] OR snack[tiab] OR snacks[tiab] OR sweet[tiab] OR sweets[tiab] OR desert[tiab] OR “ice cream”[tiab] OR biscuit*[tiab] OR cookie*[tiab] OR chocolate*[tiab] OR pizza[tiab] OR grain[tiab] OR grains[tiab] OR pasta[tiab] OR cereal[tiab] OR cereals[tiab] OR corn[tiab] OR maize[tiab] OR rice[tiab] OR wheat[tiab] OR barley[tiab] OR sorghum[tiab] OR oat[tiab] OR oats[tiab] OR oatmeal[tiab] OR rye[tiab] OR cassava[tiab] OR soybean[tiab] OR yam[tiab] OR plantain[tiab] OR millet[tiab] OR taro[tiab] OR milk[tiab] OR “low fat dairy”[tiab] OR “low-fat dairy”[tiab] OR “lowfat dairy”[tiab] OR butter[tiab] OR cheese[tiab] OR cheeses[tiab] OR margarine[tiab] OR lard[tiab] OR yogurt[tiab] OR “soybean oil”[tiab] OR “rapeseed oil”[tiab] OR “sunflower oil”[tiab] OR “olive oil”[tiab] OR “sesame oil”[tiab] OR “palm oil”[tiab] OR “coconut oil”[tiab] OR “cooking oil”[tiab] OR “vegetable oil”[tiab] OR “soya oil”[tiab] OR “peanut oil”[tiab] OR “groundnut oil”[tiab] OR fish[tiab] OR seafood[tiab] OR pollock[tiab] OR anchovy[tiab] OR tuna[tiab] OR sardine[tiab] OR mackerel[tiab] OR herring[tiab] OR cod[tiab] OR scad[tiab] OR hairtail[tiab] OR whiting[tiab] OR

squid[tiab] OR threadfin[tiab] OR menhaden[tiab] OR sprat[tiab] OR crab[tiab] OR shrimp[tiab] OR pilchard[tiab] OR

meat[tiab] OR meats[tiab] OR “lean protein”[tiab] OR beef[tiab] OR poultry[tiab] OR chicken[tiab] OR pork[tiab] OR mutton[tiab] OR goat[tiab] OR egg[tiab] OR eggs[tiab] OR fats[tiab] OR “saturated fat”[tiab] OR “unsaturated fat”[tiab] OR polyunsat*[tiab] OR poly-unsat*[tiab] OR monounsat*[tiab] OR mono-unsat*[tiab] OR transfat[tiab] OR trans-fat*[tiab] OR sodium [tiab] OR salt[tiab] OR sugar[tiab] OR sugars[tiab] OR sucrose[tiab] OR isoglucose[tiab] OR fiber[tiab] OR fibre[tiab] OR “infant formula”[tiab] OR “baby food”[tiab] OR “baby foods”[tiab] OR "infant food"[tiab] OR "infant foods"[tiab] OR “toddler formula”[tiab] OR “toddler milks”[tiab]

NOT

drug[tiab] or drugs[tiab]

Search results with the terms above on 7/3/19: n=8,261
Search results with the terms above on 1/31/20: n=258
Search results with the terms above on 6/01/20: n=205

Business Source Complete / CINAHL / EconLit / PSYCInfo Search Terms

Concept #1: TREATMENT OR INTERVENTION

TI(tax OR taxes OR taxation OR taxed OR taxing OR subsidy OR subsidies OR subsidized OR coupon OR coupons OR incentive OR incentives OR disincentive OR disincentives OR discount OR discounts OR discounted OR pricing OR priced OR “fiscal policy” OR “fiscal policies” OR voucher OR vouchers OR rebate OR rebates OR levy OR "price reduction" OR “price increase” OR "price promotion” OR “price promotions” OR “price floor” OR “price ceiling” OR “price cap” OR “price caps”) OR

AB(tax OR taxes OR taxation OR taxed OR taxing OR subsidy OR subsidies OR subsidized OR coupon OR coupons OR incentive OR incentives OR disincentive OR disincentives OR discount OR discounts OR discounted OR pricing OR priced OR “fiscal policy” OR “fiscal policies” OR voucher OR vouchers OR rebate OR rebates OR levy OR "price reduction" OR “price increase” OR "price promotion” OR “price promotions” OR “price floor” OR “price ceiling” OR “price cap” OR “price caps”)

AND

Concept #2: TARGETS FOR TREATMENT OR INTERVENTION

TI(“sugary drink” OR “sugary drinks” OR “sugary beverages” OR “sweetened beverage” OR “sweetened beverages” OR “sweetened drink” OR “sweetened drinks” OR “sugar-sweetened beverage” OR “sugar-sweetened beverages” OR “sugar-sweetened drink” OR “sugar-sweetened drinks” OR “carbonated beverage” OR “carbonated beverages” OR “carbonated-beverage” OR “carbonated-beverages” OR “carbonated drink” OR “carbonated drinks” OR “carbonated-drink” OR “carbonated-drinks” OR “high-calorie drink” OR “high-calorie drinks” OR “high calorie drink” OR “high calorie drinks” OR “high-calorie beverage” OR “high-calorie beverages” OR “high calorie beverage” OR “high calorie beverages” OR “low-calorie drink” OR “low-calorie drinks” OR “low calorie drink” OR “low calorie drinks” OR “low-calorie beverage” OR “low-calorie beverages” OR “low calorie beverage” OR “low calorie beverages” OR “soft drink” OR “soft drinks” OR “soft-drink” OR “soft-drinks” OR “fizzy drink” OR “fizzy drinks” OR “fizzy-drink” OR “fizzy-drinks” OR “energy drink” OR “energy drinks” OR “energy-drink” OR “energy-drinks” OR “fruit drink” OR “fruit drinks” OR “fruit-drink” OR “fruit-drinks” OR “sports drink” OR “sports drinks” OR “sports-drink” OR “sports-drinks” OR tea OR teas OR coffee OR coffees OR soda OR sodas OR cola OR colas OR “flavored water” OR “vitamin water” OR SSB* OR “soda pop” OR “chocolate milk” OR “flavored milk” OR juice OR syrup* OR “healthy food” OR “healthy foods” OR “unhealthy food” OR “unhealthy foods” OR “nutritious foods” OR “junk food” OR “junk foods” OR “energy dense food” OR “high-calorie food” OR “highcalorie food” OR “high calorie food” OR “energy dense foods” OR “high-calorie foods” OR “highcalorie foods” OR “high calorie foods” OR “low-calorie food” OR “lowcalorie food” OR “low calorie food” OR “low-calorie foods” OR “lowcalorie foods” OR “low calorie foods” OR "highenergy food" OR "high energy food" OR "high-energy food" OR "highenergy foods" OR "high energy foods" OR "high-energy foods" OR "lowenergy food" OR "low energy food" OR "low-energy food" OR "lowenergy foods" OR "low energy foods" OR "low-energy foods" OR “low-fat” OR “low fat” OR “lowfat” OR “fatty food” OR “fatty foods” OR “fattening food” OR “fattening-food” OR “salty food” OR “salty-food” OR “processed food” OR “fattening foods” OR “fattening-foods” OR “salty foods” OR “salty-foods” OR “processed foods” OR “takeout food” OR “take-out food” OR “takeaway food” OR “take-away food” OR “takeout foods” OR “take-out foods” OR “takeaway foods” OR “take-away foods” OR “fast food” OR “fast-food” OR “fast foods” OR “fast-foods” OR fastfood OR fruit OR fruits OR vegetable OR vegetables OR legumes OR pulses OR beans OR lentils OR peas OR peanut* OR salad OR potato OR potatoes OR burger OR candy OR candies OR confectionar* OR chips OR snack OR snacks OR sweet OR sweets OR desert OR “ice cream” OR biscuit* OR cookie* OR chocolate* OR pizza OR chips OR grain OR grains OR pasta OR cereal OR cereals OR corn OR maize OR rice OR wheat OR barley OR sorghum OR oat OR oats OR oatmeal OR rye OR cassava OR soybean OR yam OR plantain OR millet OR taro OR milk OR “low fat dairy” OR “low-fat dairy” OR “lowfat dairy” OR butter OR cheese OR cheeses OR margarine OR lard OR yogurt OR “soybean oil” OR “rapeseed oil” OR “sunflower oil” OR “olive oil” OR “sesame oil” OR “palm oil” OR “coconut oil” OR “cooking oil” OR “vegetable oil” OR “soya oil” OR “peanut oil” OR “groundnut oil” OR fish OR fishes OR seafood OR pollock OR anchovy OR tuna OR sardine OR mackerel OR herring OR cod OR scad OR hairtail OR whiting OR squid OR threadfin OR menhaden OR sprat OR crab OR shrimp OR pilchard OR meat OR meats OR “lean protein” OR beef OR poultry OR chicken OR pork OR mutton OR goat OR egg OR eggs OR fats OR “saturated fat” OR “unsaturated fat” OR polyunsat* OR poly-unsat* OR monounsat* OR mono-unsat* OR transfat OR trans-fat* OR sodium OR salt OR sugar OR sugars OR sucrose OR isoglucose OR fiber

© 2022 Andreyeva T et al. *JAMA Network Open.*

OR fibre OR “infant formula” OR “baby food” OR “baby foods” OR "infant food" OR "infant foods" OR “toddler formula” OR “toddler milks”) OR

AB(“sugary drink” OR “sugary drinks” OR “sugary beverages” OR “sweetened beverage” OR “sweetened beverages” OR “sweetened drink” OR “sweetened drinks” OR “sugar-sweetened beverage” OR “sugar-sweetened beverages” OR “sugar-sweetened drink” OR “sugar-sweetened drinks” OR “carbonated beverage” OR “carbonated beverages” OR “carbonated-beverage” OR “carbonated-beverages” OR “carbonated drink” OR “carbonated drinks” OR “carbonated-drink” OR “carbonated-drinks” OR “high-calorie drink” OR “high-calorie drinks” OR “high calorie drink” OR “high calorie drinks” OR “high-calorie beverage” OR “high-calorie beverages” OR “high calorie beverage” OR “high calorie beverages” OR “low-calorie drink” OR “low-calorie drinks” OR “low calorie drink” OR “low calorie drinks” OR “low-calorie beverage” OR “low-calorie beverages” OR “low calorie beverage” OR “low calorie beverages” OR “soft drink” OR “soft drinks” OR “soft-drink” OR “soft-drinks” OR “fizzy drink” OR “fizzy drinks” OR “fizzy-drink” OR “fizzy-drinks” OR “energy drink” OR “energy drinks” OR “energy-drink” OR “energy-drinks” OR “fruit drink” OR “fruit drinks” OR “fruit-drink” OR “fruit-drinks” OR “sports drink” OR “sports drinks” OR “sports-drink” OR “sports-drinks” OR tea OR teas OR coffee OR coffees OR soda OR sodas OR cola OR colas OR “flavored water” OR “vitamin water” OR SSB* OR “soda pop” OR “chocolate milk” OR “flavored milk” OR juice OR syrup* OR
“healthy food” OR “healthy foods” OR “unhealthy food” OR “unhealthy foods” OR “nutritious foods” OR “junk food” OR “junk foods” OR “energy dense food” OR “high-calorie food” OR “highcalorie food” OR “high calorie food” OR “energy dense foods” OR “high-calorie foods” OR “highcalorie foods” OR “high calorie foods” OR “low-calorie food” OR “lowcalorie food” OR “low calorie food” OR “low-calorie foods” OR “lowcalorie foods” OR “low calorie foods” OR "highenergy food" OR "high energy food" OR "high-energy food" OR "highenergy foods" OR "high energy foods" OR "high-energy foods" OR "lowenergy food" OR "low energy food" OR "low-energy food" OR "lowenergy foods" OR "low energy foods" OR "low-energy foods" OR “low-fat” OR “low fat” OR “lowfat” OR “fatty food” OR “fatty foods” OR “fattening food” OR “fattening-food” OR “salty food” OR “salty-food” OR “processed food” OR “fattening foods” OR “fattening-foods” OR “salty foods” OR “salty-foods” OR “processed foods” OR “takeout food” OR “take-out food” OR “takeaway food” OR “take-away food” OR “takeout foods” OR “take-out foods” OR “takeaway foods” OR “take-away foods” OR “fast food” OR “fast-food” OR “fast foods” OR “fast-foods” OR fastfood OR fruit OR fruits OR vegetable OR vegetables OR legumes OR pulses OR beans OR lentils OR peas OR peanut* OR salad OR potato OR potatoes OR burger OR candy OR candies OR confectionar* OR chips OR snack OR snacks OR sweet OR sweets OR desert OR “ice cream” OR biscuit* OR cookie* OR chocolate* OR pizza OR chips OR grain OR grains OR pasta OR cereal OR cereals OR corn OR maize OR rice OR wheat OR barley OR sorghum OR oat OR oats OR oatmeal OR rye OR cassava OR soybean OR yam OR plantain OR millet OR taro OR milk OR “low fat dairy” OR “low-fat dairy” OR “lowfat dairy” OR butter OR cheese OR cheeses OR margarine OR lard OR yogurt OR “soybean oil” OR “rapeseed oil” OR “sunflower oil” OR “olive oil” OR “sesame oil” OR “palm oil” OR “coconut oil” OR “cooking oil” OR “vegetable oil” OR “soya oil” OR “peanut oil” OR “groundnut oil” OR fish OR fishes OR seafood OR pollock OR anchovy OR tuna OR sardine OR mackerel OR herring OR cod OR scad OR hairtail OR whiting OR squid OR threadfin OR menhaden OR sprat OR crab OR shrimp OR

© 2022 Andreyeva T et al. *JAMA Network Open.*

pilchard OR meat OR meats OR “lean protein” OR beef OR poultry OR chicken OR pork OR mutton OR goat OR egg OR eggs OR fats OR “saturated fat” OR “unsaturated fat” OR polyunsat* OR poly-unsat* OR monounsat* OR mono-unsat* OR transfat OR trans-fat* OR sodium OR salt OR sugar OR sugars OR sucrose OR isoglucose OR fiber OR fibre OR “infant formula” OR “baby food” OR “baby foods” OR "infant food" OR "infant foods" OR “toddler formula” OR “toddler milks”)

NOT

(TI(drug OR drugs) OR AB(drug OR drugs))

LIMIT TO: Academic Journals

Search results with the terms and limitations above on 7/3/19:
Business Source Complete: n=3,816
CINAHL: 842
EconLit: n=2,830
Proquest Dissertations & Theses: n=8,404
PSYCInfo: n=1,107

Search results with the terms and limitations above on 1/31/20:
Business Source Complete: n=133
CINAHL: 172
EconLit: n=153
Proquest Dissertations & Theses: n=121
PSYCInfo: n=60

Search results with the terms and limitations above on 6/1/20:
Business Source Complete: n=57
CINAHL: 49
EconLit: n=13
Proquest Dissertations & Theses: n=20
PSYCInfo: n=7

Cochrane Search Terms

Concept #1: TREATMENT OR INTERVENTION

((tax OR taxes OR taxation OR taxed OR taxing OR subsidy OR subsidies OR subsidized OR coupon OR coupons OR incentive OR incentives OR disincentive OR disincentives OR discount OR discounts OR discounted OR pricing OR priced OR “price promotion” OR “price promotions” OR “price floor” OR “price ceiling” OR “price cap” OR “price caps” OR “pricing policy” OR

© 2022 Andreyeva T et al. *JAMA Network Open.*

“pricing policies” OR “fiscal policy” OR “fiscal policies” OR “price reduction” OR “price increase” OR voucher OR vouchers OR rebate OR rebates OR levy OR “price response”)):ti,ab,kw

AND

Concept #2: TARGETS FOR TREATMENT OR INTERVENTION

((“sugary drink” OR “sugary drinks” OR “sugary beverages” OR “sweetened beverage” OR “sweetened beverages” OR “sweetened drink” OR “sweetened drinks” OR “sugar-sweetened beverage” OR “sugar-sweetened beverages” OR “sugar-sweetened drink” OR “sugar-sweetened drinks” OR “carbonated beverage” OR “carbonated beverages” OR “carbonated-beverage” OR “carbonated-beverages” OR “carbonated drink” OR “carbonated drinks” OR “carbonated-drink” OR “carbonated-drinks” OR “high-calorie drink” OR “high-calorie drinks” OR “high calorie drink” OR “high calorie drinks” OR “high-calorie beverage” OR “high-calorie beverages” OR “high calorie beverage” OR “high calorie beverages” OR “low-calorie drink” OR “low-calorie drinks” OR “low calorie drink” OR “low calorie drinks” OR “low-calorie beverage” OR “low-calorie beverages” OR “low calorie beverage” OR “low calorie beverages” OR “soft drink” OR “soft drinks” OR “soft-drink” OR “soft-drinks” OR “fizzy drink” OR “fizzy drinks” OR “fizzy-drink” OR “fizzy-drinks” OR “energy drink” OR “energy drinks” OR “energy-drink” OR “energy-drinks” OR “fruit drink” OR “fruit drinks” OR “fruit-drink” OR “fruit-drinks” OR “sports drink” OR “sports drinks” OR “sports-drink” OR “sports-drinks” OR tea OR teas OR coffee OR coffees OR soda OR sodas OR cola OR colas OR “flavored water” OR “vitamin water” OR SSB OR SSBs OR “soda pop” OR “chocolate milk” OR “flavored milk” OR juice OR syrup OR syrups OR “healthy food” OR “healthy foods” OR “unhealthy food” OR “unhealthy foods” OR “nutritious foods” OR “junk food” OR “junk foods” OR “energy dense food” OR “high-calorie food” OR “highcalorie food” OR “high calorie food” OR “energy dense foods” OR “high-calorie foods” OR “highcalorie foods” OR “high calorie foods” OR “low-calorie food” OR “lowcalorie food” OR “low calorie food” OR “low-calorie foods” OR “lowcalorie foods” OR “low calorie foods” OR "highenergy food" OR "high energy food" OR "high-energy food" OR "highenergy foods" OR "high energy foods" OR "high-energy foods" OR "lowenergy food" OR "low energy food" OR "low-energy food" OR "lowenergy foods" OR "low energy foods" OR "low-energy foods" OR “low-fat” OR “low fat” OR “lowfat” OR “fatty food” OR “fatty foods” OR “fattening food” OR “fattening-food” OR “salty food” OR “salty-food” OR “processed food” OR “fattening foods” OR “fattening-foods” OR “salty foods” OR “salty-foods” OR “processed foods” OR “takeout food” OR “take-out food” OR “takeaway food” OR “take-away food” OR “takeout foods” OR “take-out foods” OR “takeaway foods” OR “take-away foods” OR “fast food” OR “fast-food” OR “fast foods” OR “fast-foods” OR fastfood OR fruit OR fruits OR vegetable OR vegetables OR legumes OR pulses OR beans OR lentils OR peas OR peanut OR peanuts OR salad OR potato OR potatoes OR burger OR candy OR candies OR confectionary OR confectionaries OR chips OR snack OR snacks OR sweet OR sweets OR desert OR “ice cream” OR biscuit OR biscuits OR cookie OR cookies OR chocolate OR chocolates OR pizza OR chips OR grain OR grains OR pasta OR cereal OR cereals OR corn OR maize OR rice OR wheat OR barley OR sorghum OR oat OR oats OR oatmeal OR rye OR cassava OR soybean OR yam OR plantain OR millet OR taro OR milk OR “low fat dairy” OR “low-fat dairy” OR “lowfat dairy” OR butter OR cheese OR cheeses OR margarine OR lard OR yogurt OR

© 2022 Andreyeva T et al. *JAMA Network Open.*

“soybean oil” OR “rapeseed oil” OR “sunflower oil” OR “olive oil” OR “sesame oil” OR “palm oil” OR “coconut oil” OR “cooking oil” OR “vegetable oil” OR “soya oil” OR “peanut oil” OR “groundnut oil” OR fish OR fishes OR seafood OR pollock OR anchovy OR tuna OR sardine OR mackerel OR herring OR cod OR scad OR hairtail OR whiting OR squid OR threadfin OR menhaden OR sprat OR crab OR shrimp OR pilchard OR meat OR meats OR “lean protein” OR beef OR poultry OR chicken OR pork OR mutton OR goat OR egg OR eggs OR fats OR “saturated fat” OR “unsaturated fat” OR polyunsaturated OR poly-unsaturated OR monounsaturated OR mono-unsaturated OR transfat OR trans-fats OR sodium OR salt OR sugar OR sugars OR sucrose OR isoglucose OR fiber OR fibre OR “infant formula” OR “baby food” OR “baby foods” OR "infant food" OR "infant foods" OR “toddler formula” OR “toddler milks”)):ti,ab,kw

NOT (drug OR drugs):ti,ab,kw

Search results in Cochrane CENTRAL with the terms above on 7/3/19: n=335
Search results in Cochrane CENTRAL with the terms above on 1/31/20: n=41
Search results in Cochrane CENTRAL with the terms above on 6/1/20: n=24
Search results in Cochrane CDSR with the terms above on 7/3/19: n=6
Search results in Cochrane CDSR with the terms above on 1/31/20: n=1
Search results in Cochrane CDSR with the terms above on 6/1/20: n=1

SCOPUS Search Terms

Concept #1: TREATMENT OR INTERVENTION

TITLE-ABS (tax OR taxes OR taxation OR taxed OR taxing OR subsidy OR subsidies OR subsidized OR coupon OR coupons OR incentive OR incentives OR disincentive OR disincentives OR discount OR discounts OR discounted OR pricing OR priced OR "fiscal policy" OR "fiscal policies" OR voucher OR vouchers OR rebate OR rebates OR levy OR "price reduction" OR “price increase” OR "price promotion" OR "price promotions" OR "price floor" OR "price ceiling" OR "price cap" OR "price caps")

AND
Concept #2: TARGETS FOR TREATMENT OR INTERVENTION

TITLE-ABS ("sugary drink" OR "sugary drinks" OR “sugary beverages” OR "sweetened beverage" OR "sweetened beverages" OR "sweetened drink" OR "sweetened drinks" OR "sugar-sweetened beverage" OR "sugar-sweetened beverages" OR "sugar-sweetened drink" OR "sugar-sweetened drinks" OR "carbonated beverage" OR "carbonated beverages" OR "carbonated-beverage" OR "carbonated-beverages" OR "carbonated drink" OR "carbonated drinks" OR "carbonated-drink" OR "carbonated-drinks" OR "high-calorie drink" OR "high-calorie drinks" OR "high calorie drink" OR "high calorie drinks" OR "high-calorie beverage" OR "high-calorie beverages" OR "high calorie beverage" OR "high calorie beverages" OR "low-calorie drink" OR "low-calorie drinks" OR "low calorie drink" OR "low calorie drinks"

© 2022 Andreyeva T et al. *JAMA Network Open.*

OR "low-calorie beverage" OR "low-calorie beverages" OR "low calorie beverage" OR "low calorie beverages" OR "soft drink" OR "soft drinks" OR "soft-drink" OR "soft-drinks" OR "fizzy drink" OR "fizzy drinks" OR "fizzy-drink" OR "fizzy-drinks" OR "energy drink" OR "energy drinks" OR "energy-drink" OR "energy-drinks" OR "fruit drink" OR "fruit drinks" OR "fruit-drink" OR "fruit-drinks" OR "sports drink" OR "sports drinks" OR "sports-drink" OR "sports-drinks" OR tea OR teas OR coffee OR coffees OR soda OR sodas OR cola OR colas OR "flavored water" OR "vitamin water" OR ssb* OR "soda pop" OR "chocolate milk" OR "flavored milk" OR juice OR syrup* OR "healthy food" OR "healthy foods" OR "unhealthy food" OR "unhealthy foods" OR “nutritious foods” OR "junk food" OR "junk foods" OR "energy dense food" OR "high-calorie food" OR "highcalorie food" OR "high calorie food" OR "energy dense foods" OR "high-calorie foods" OR "highcalorie foods" OR "high calorie foods" OR "low-calorie food" OR "lowcalorie food" OR "low calorie food" OR "low-calorie foods" OR "lowcalorie foods" OR "low calorie foods" OR "highenergy food" OR "high energy food" OR "high-energy food" OR "highenergy foods" OR "high energy foods" OR "high-energy foods" OR "lowenergy food" OR "low energy food" OR "low-energy food" OR "lowenergy foods" OR "low energy foods" OR "low-energy foods" OR "low-fat" OR "low fat" OR "lowfat" OR "fatty food" OR "fatty foods" OR "fattening food" OR "fattening-food" OR "salty food" OR "salty-food" OR "processed food" OR "fattening foods" OR "fattening-foods" OR "salty foods" OR "salty-foods" OR "processed foods" OR "takeout food" OR "take-out food" OR "takeaway food" OR "take-away food" OR "takeout foods" OR "take-out foods" OR "takeaway foods" OR "take-away foods" OR "fast food" OR "fast-food" OR "fast foods" OR "fast-foods" OR fastfood OR fruit OR fruits OR vegetable OR vegetables OR legumes OR pulses OR beans OR lentils OR peas OR peanut* OR salad OR potato OR potatoes OR burger OR candy OR candies OR confectionar* OR chips OR snack OR snacks OR sweet OR sweets OR desert OR "ice cream" OR biscuit* OR cookie* OR chocolate* OR pizza OR chips OR grain OR grains OR pasta OR cereal OR cereals OR corn OR maize OR rice OR wheat OR barley OR sorghum OR oat OR oats OR oatmeal OR rye OR cassava OR soybean OR yam OR plantain OR millet OR taro OR milk OR "low fat dairy" OR "low-fat dairy" OR "lowfat dairy" OR butter OR cheese OR cheeses OR margarine OR lard OR yogurt OR "soybean oil" OR "rapeseed oil" OR "sunflower oil" OR "olive oil" OR "sesame oil" OR "palm oil" OR "coconut oil" OR "cooking oil" OR "vegetable oil" OR "soya oil" OR "peanut oil" OR "groundnut oil" OR fish OR fishes OR seafood OR pollock OR anchovy OR tuna OR sardine OR mackerel OR herring OR cod OR scad OR hairtail OR whiting OR squid OR threadfin OR menhaden OR sprat OR crab OR shrimp OR pilchard OR meat OR meats OR "lean protein" OR beef OR poultry OR chicken OR pork OR mutton OR goat OR egg OR eggs OR fats OR "saturated fat" OR "unsaturated fat" OR polyunsat* OR poly-unsat* OR monounsat* OR mono-unsat* OR transfat OR trans-fat* OR sodium OR salt OR sugar OR sugars OR sucrose OR isoglucose OR fiber OR fibre OR "infant formula" OR "baby food" OR "baby foods" OR "infant food" OR "infant foods" OR "toddler formula" OR "toddler milks")

AND NOT TITLE-ABS (drug OR drugs)

AND (LIMIT-TO (SRCTYPE , "j"))

© 2022 Andreyeva T et al. *JAMA Network Open.*

Search results in SCOPUS with the terms above on 7/3/19: n=15,814
Search results in SCOPUS with the terms above on 1/31/20: n=1,542
Search results in SCOPUS with the terms above on 6/1/20: n=651

Standard Grey Literature Search Terms

Concept #1: TREATMENT OR INTERVENTION

(tax* OR subsid* OR coupon* OR incentive* OR discount* OR pricing OR “price promotion” OR “pricing policy” OR “fiscal policy” OR voucher* OR rebate* OR levy)

AND

Concept #2: TARGETS FOR TREATMENT OR INTERVENTION

(“sugary drinks” OR “sugar-sweetened beverages” OR “sweetened beverages” OR “soft drinks” OR soda OR “high-sugar” OR “healthy food” OR “nutritious food” OR “fatty food” OR “high-calorie” OR “energy-dense” OR “junk food” OR “unhealthy food” OR “low fat” OR sugar OR “processed food” OR “fast food” OR fruit OR vegetables OR grain OR milk OR meat OR snack OR “lean protein” OR fats OR “saturated fat” OR “infant formula” OR sodium)

Search results with the terms above on 8/17/19:
WHO’s International Clinical Trials Registry Platform: n=9
PDQ-Evidence for Informed Health Policymaking: n=56
Healthevidence.org: n=35
WHO Global Index Medicus: n=63
WorldWideScience: n=1,318

Search results with the terms above on 1/31/20:
WHO’s International Clinical Trials Registry Platform: n=1
PDQ-Evidence for Informed Health Policymaking: n=0
Healthevidence.org: n=50
WHO Global Index Medicus: n=6
WorldWideScience: n=30

Search results with the terms above on 6/1/20:
WHO’s International Clinical Trials Registry Platform: n=0
PDQ-Evidence for Informed Health Policymaking: n=0
Healthevidence.org: n=17
WHO Global Index Medicus: n=0
WorldWideScience: n=0

© 2022 Andreyeva T et al. *JAMA Network Open.*

Search results with the above terms and limited to the last three years and the top 500 most relevant results:
National Bureau of Economic Research: n=500
New papers added on 1/31 with the same search terms: 548
New papers added on 6/1 with the same search terms: 33

Search results with the above terms and limited to the top 500 most relevant results:
EconPapers: n=500
New papers added on 1/31 with the same search terms: 500
New papers added on 6/1 with the same search terms: 37

Search results with the above terms and limited to the top 100 most relevant results:
Google Scholar: n=100
New papers added on 1/31 with the same search terms: 100
New papers added on 6/1 with the same search terms: 94

Search results with the second concept only on 8/17/19:
Directory of Open Access Journals: n=250
New papers added on 1/31 with the same search terms: 65
New papers added on 6/1 with the same search terms: 20

Highly Simplified Grey Literature Search Terms

"tax*" OR "subsid*" OR "coupon*" OR "incentive*" OR "discount*" OR "pricing"

Free-text Search results with the terms above on 8/17/19:
EPPI-Centre Database of Promoting Health Effectiveness Reviews (DoPHER): n=359
EPPI-Centre Trials Register of Promoting Health Interventions (TRoPHI): n=324

Free-text Search results with the terms above on 6/1/20:
EPPI-Centre Database of Promoting Health Effectiveness Reviews (DoPHER): n=25
EPPI-Centre Trials Register of Promoting Health Interventions (TRoPHI): n=2

Two Word Grey Literature AND Combo Search Terms
tax and beverages
tax and drinks
tax and food
incentive and beverages
incentive and drinks
incentive and food
subsidy and beverages
subsidy and drinks
subsidy and food

© 2022 Andreyeva T et al. *JAMA Network Open.*

pricing and beverages
pricing and drinks
pricing and food
"fiscal policy" and beverages
"fiscal policy" and drinks
"fiscal policy" and food

Search results with the terms above on 8/17/19:
SSRN eLibrary: n=1,226
New papers added on 1/31 with the same search terms: 54
New papers added on 6/1 with the same search terms: 0

Hand-Screened References

References from systematic reviews identified using the search strategy above:
n=2,109

References from papers meeting the inclusion criteria
n=90

Hand-Screened Government Websites

Reports from the World Health Organization website
n=30
Reports from the United States Department of Agriculture website
n=8
Reports from foreign government websites
n=46
Reports from other government websites
n=3

We used Covidence (https://app.covidence.org) and Mendeley (https://www.mendeley.com) as the reference management software to manage the review.

© 2022 Andreyeva T et al. *JAMA Network Open.*

eAppendix 2. Computation of Price Elasticity of Demand and Cross-Price Elasticity of Demand Measures

In this review, in addition to examining effect size estimates of outcomes for changes in demand (e.g., sales and consumption), we also convert each demand estimate into a common metric measure known as price elasticity of demand. The price elasticity of demand measure is calculated as a percentage change in demand (sales or consumption) over a percentage change in price. Cross price elasticity measures substitution effects and is calculated as a percentage change in demand of SSB substitute beverages over a percentage change in SSB prices due to SSB taxes. SSB substitute beverages are untaxed beverages or bottled water. To estimate the price elasticity of demand for taxed beverages, an overall measure of post-tax percentage change in price was calculated for each taxing jurisdiction.

Baseline prices of taxed beverages and estimates of tax pass-through were extracted from the studies that evaluated tax impacts on prices. Jurisdiction-specific pass-through rates were estimated in a meta-analysis of studies by jurisdiction. Percentage change in price for each jurisdiction was calculated by multiplying an estimated pass-through rate by the tax rate and dividing by the median baseline price. Elasticities and their confidence intervals were then computed from relative changes in demand by dividing estimates of the percentage change in demand and corresponding confidence intervals by the jurisdiction-level percentage change in price.

© 2022 Andreyeva T et al. *JAMA Network Open.*

eTable 1. Quality Assessment of Fiscal Policy Studies

	Question	Scoring System (Yes = 1.0, Partial = 0.5, No = 0)
Control group	1. Does the study compare outcomes in an appropriate control group?	Yes = Control group (e.g., not impacted by policy) or counterfactual used.
		No = Uncontrolled study (no control group or counterfactual).
Measures	2. Does the study measure outcomes objectively?	Yes = Objective measures: sales (scanner data), purchases (receipts of purchases), prices (scanner data), measured body weight/height, health measurements (e.g., blood pressure), 24-hour dietary intake recall, government/ administrative records.
		Partial = Data recorded by raters or households, e.g., households recording purchases without receipts.
		No = Participant self-reported data: self-reported BMI, food frequency surveys, other surveys with self-reported data.
Follow Up	3. Does the study assess outcomes in the same people/ stores before and after the intervention?	Yes = Longitudinal design: same people/households/stores at baseline and follow-up.
		No = Not the same people/households/stores, e.g., repeated cross-sections or longitudinal data with high attrition rates (>25%).
Duration	4. How many data points does the study examine before and after the intervention?	Yes = The study has ≥3 data points before and after the intervention.
		Partial = The study meets the above criteria for either a pre- or post-intervention, but not both.
		No = The study has 1-2 observations per pre- and post-assessment.
Sample	5. Does the study use a representative sample of the population affected by the policy?	Yes = Representative data of the affected population, e.g., random population sampling.
		Partial = Somewhat representative of the affected population, includes scanner household consumer panels.
		No = Not fully representative or convenience sample, e.g., limited to only one store type or one chain, select list of products or brands or group of customers.
Sample	6. Does the study have a sufficient sample size?	Yes = Sample size or power analyses provided and shows sufficient sample size.
		No = No information provided or individual sample sizes appear insufficient; e.g., n<500 individuals per site/time period or store audits n<50 per site/time period.

© 2022 Andreyeva T et al. *JAMA Network Open.*

Confounding	7. Does the study adjust adequately for likely confounders?	Yes = If an ITS then it adjusted/removed the effect of existing time trends, seasonality, and economic fluctuations. If different people are in comparison groups, then also adjusts for demographic differences, such as age and socioeconomic status.
		Partial = Some of these factors were adjusted for.
		No = None of these factors were adjusted for.
**Total Score:** Sum scores on all measures (range 0 to 7)<br>Low quality score ≤3.0<br>Medium quality score 3.5-4.5<br>High quality score ≥5.0		

© 2022 Andreyeva T et al. *JAMA Network Open.*

eTable 2. Characteristics of Studies

Source	Tax jurisdiction	Tax type (taxed beverages)[a]	Outcome(s) included	Study design	Data source/type	Population[b]	Quality score	Peer-reviewed[c]
Aguilar et al,[29] 2019	Mexico	1 peso per liter excise tax (SSBs)	Price changes, SSB sales, sales/ substitution	ITS with counterfactual	Scanner: Kantar Worldpanel	All	High	No[c]
Alsukait et al,[66] 2020	Saudi Arabia	50% ad valorem excise tax (carbonated SSBs) 100% ad valorem excise tax (energy drinks)	Price changes	Before and after with counterfactual	Administrative: General Authority of Statistics	All	Medium	Yes
			SSB sales, sales/ substitution	CBA	Database: Euromonitor Passport		Low	
Alvarado et al,[62] 2017	Barbados	10% ad valorem excise tax (SSBs)	Price changes	Uncontrolled before and after	Scanner: one grocery chain	All	Low	Yes
Alvarado et al,[63] 2019	Barbados	10% ad valorem excise tax (SSBs)	SSB sales, sales/ substitution	ITS with counterfactual	Scanner: one grocery chain	All	High	Yes
Andalón and Gibson,[41] 2017	Mexico	1 peso per liter excise tax (SSBs)	Price changes	Uncontrolled before and after	Administrative: INEGI	All	Medium	No
			SSB sales	Uncontrolled before and after	Household survey: ENIGH		Low	

© 2022 Andreyeva T et al. *JAMA Network Open.*

Arteaga et al,[30] 2017	Mexico	1 peso per liter excise tax (SSBs)	SSB sales	Uncontrolled ITS	Industry survey: EMIM	All	Low	No[c]
Baskin and Coary,[76] 2019	Philadelphia, US	$0.015 per ounce excise tax (SSBs, ASBs)	SSB sales, sales/ substitution UC (cross-border)	CBA	Scanner: one grocery chain	All	Medium	Yes
Berardi et al,[53] 2016	France	€0.0716 per liter excise tax (SSBs, ASBs)	Price changes	Before and after with counterfactual	Scanner: Prixing drive-through price lists	All	High	Yes
Bergman and Hansen,[60] 2019	Denmark	Up to DKK 1.65 per liter excise tax (SSBs)	Price changes	Uncontrolled ITS	Administrative: Statistics Denmark	All	Medium	Yes
Bollinger and Sexton,[82] 2018	Berkeley, US	$0.01 per ounce excise tax (SSBs)	Price changes, SSB sales	CBA	Scanner: Nielsen retail	All	High	No
Bonilla-Chacin et al,[42] 2016	Mexico	1 peso per liter excise tax (SSBs)	SSB sales, sales/ substitution	Uncontrolled before and after	Household survey: ENIGH	All	Low	No
			Price changes	Uncontrolled before and after	Administrative: INEGI		Medium	
Breeze et al,[113] 2018	Sheffield, UK	£0.20 beverage levy (SSBs)	SSB sales, sales/ substitution	Uncontrolled ITS	Scanner: café sales	All	Medium	Yes
Campos-Vázquez and Medina-	Mexico	1 peso per liter excise tax (SSBs)	Price changes	Uncontrolled ITS	Administrative: Federal Consumer	All	Medium	Yes

© 2022 Andreyeva T et al. *JAMA Network Open.*

Cortina,[31] 2019					Protection Agency			
Capacci et al,[54] 2019	France	€0.0716 per liter excise tax (SSBs, ASBs)	Price changes	CBA	Administrative: Italian and French National Institutes of Statistics Scanner: Kantar WorldPanel France, GfK Italy	All	High	Yes
			SSB sales, sales/ substitution	CBA	Scanner: Kantar WorldPanel France, GfK Italy; Survey: Italian Household Expenditure Survey		Medium	
Caro et al,[57] 2018	Chile	10% (<6.25g sugar/100 ml) and 18% (≥6.25 g sugar/100ml) ad valorem tax (SSBs, ASBs)	Price changes, SSB sales, sales/ substitution	ITS with counterfactual	Scanner: Kantar World Panel Chile	All	High	Yes

© 2022 Andreyeva T et al. *JAMA Network Open.*

Cawley and Frisvold,[81] 2017	Berkeley, US	$0.01 per ounce excise tax (SSBs)	Price changes	CBA	Primary: store audits	All	Medium	Yes
Cawley et al,[68] 2018	Philadelphia, US	$0.015 per ounce excise tax (SSBs, ASBs)	Price changes	CBA	Primary: store audits	All	Medium	Yes
Cawley et al,[112] 2018	Boulder, US	$0.02 per ounce excise tax (SSBs)	Price changes	CBA	Primary: store and limited-service restaurant audits, webscraping: restaurant menus	All	High	No[c]
Cawley et al,[70] 2019	Philadelphia, US	$0.015 per ounce excise tax (SSBs, ASBs)	SSB consumption, consumption/ substitution	CBA	Primary: longitudinal household survey	Adults, children	Low	Yes
			SSB sales, sales/ substitution, UC (cross-border)	CBA	Primary: household survey	Households with children	Low	Yes
Cawley et al,[69] 2019	Philadelphia PA, Seattle WA, San Francisco/ Oakland CA	$0.01 to $0.0175 per ounce excise tax (SSBs, ASBs and SSBs)	SSB sales, sales/ substitution	CBA	Scanner: InfoScout Consumer Panel	All	High	No[c]
Cawley et al,[80] 2020	Philadelphia, US	$0.015 per ounce excise	Price changes	CBA	Primary: store audits	All	Medium	Yes

© 2022 Andreyeva T et al. *JAMA Network Open.*

		tax (SSBs, ASBs)						
Cawley et al,[99] 2020	Oakland, US	$0.01 per ounce excise tax (SSBs)	Price changes	CBA	Primary: store audits	All	Medium	Yes
			SSB sales, sales/ substitution, UC (cross-border)	CBA	Primary: consumer surveys	Adults, children	Low	
			SSB consumption, consumption/ substitution, UC (cross-border)	CBA	Primary: longitudinal household survey			
Chu et al,[48] 2020	United Kingdom	£0.24 per liter (≥8 g sugar/100 ml) and £0.18 per liter (5-8 g sugar/100 ml) excise tax (SSBs)	Product change	Uncontrolled before and after	Web scraping: online tool my Supermarket, websites of 9 major grocery chains	All	Low	Yes
Coary and Baskin,[77] 2018	Philadelphia, US	$0.015 per ounce excise tax (SSBs, ASBs)	Price changes	CBA	Primary: store audits	All	Low	Yes
Colantuoni and Rojas,[93] 2015	Ohio and Maine, US	5.5% (ME) and 5.0% (OH) state sales tax (soft drinks)	Price changes, SSB sales	CBA	Scanner: IRI retail	All	High	Yes

© 2022 Andreyeva T et al. *JAMA Network Open.*

Colchero et al,[32] 2015	Mexico	1 peso per liter excise tax (SSBs)	Price changes	Uncontrolled ITS	Administrative: INEGI	All	High	Yes
Colchero et al,[33] 2016	Mexico	1 peso per liter excise tax (SSBs)	SSB sales, sales/ substitution	Uncontrolled ITS	Industry survey: EMIM	All	Medium	Yes
Colchero et al,[34] 2016	Mexico	1 peso per liter excise tax (SSBs)	SSB sales, sales/ substitution	ITS with counterfactual	Scanner: Nielsen Mexico Consumer Panel Services	All	High	Yes
Colchero et al,[35] 2017	Mexico	1 peso per liter excise tax (SSBs)	SSB sales, sales/ substitution	ITS with counterfactual	Scanner: Nielsen Mexico Consumer Panel Services	All	High	Yes
Colchero et al,[36] 2017	Mexico	1 peso per liter excise tax (SSBs)	Price changes	Uncontrolled before and after	Primary: store audits	All	Low	Yes
Colchero et al,[37] 2017	Mexico	1 peso per liter excise tax (SSBs)	SSB sales, sales/ substitution	Before and after with counterfactual	Household survey: ENIGH	All	Medium	Yes
Cornelsen et al,[114] 2017	Restaurant chain, UK	£0.10 levy (SSBs)	SSB sales, sales/ substitution	Uncontrolled ITS	Scanner: one restaurant chain	All	Low	Yes
Cuadrado et al,[58] 2020	Chile	10% (<6.25g sugar/100 ml) and 18% (≥6.25 g sugar/100ml	Price changes	Uncontrolled ITS	Administrative: Chilean National Institute of Statistics	All	Medium	Yes

© 2022 Andreyeva T et al. *JAMA Network Open.*

		) ad valorem ad valorem excise tax (SSBs, ASBs)						
Dubois et al,[46] 2020	United Kingdom	£0.24 per liter (≥8 g sugar/100 ml) and £0.18 per liter (5-8 g sugar/100 ml) excise tax (SSBs)	Price changes	Uncontrolled ITS	Scanner: Kantar Worldpanel	All	High	No[c]
ECSIPC,[56] 2014	Denmark	DKK 0.82 per iL (>0.5g sugar/100 ml) and DKK 0.295/il (<0.5g/100 ml) (excise tax (soft drinks, juice)	Price changes, SSB sales	Uncontrolled before and after	Database: Euromonitor Passport	All	Low	No
	Finland	€0.11-0.22 per liter excise duty tax (SSBs, ASBs)						
	France	€0.0716 per liter excise tax (SSBs, ASBs)						

© 2022 Andreyeva T et al. *JAMA Network Open.*

	Hungary	7 HUF per liter (>8g sugar/100ml), 250 HUF per liter (energy drinks) excise tax						
Etilé et al,[55] 2018	France	€0.0716 per liter excise tax (SSBs, ASBs)	Price changes	Before and after with counterfactual	Scanner: Kantar Worldpanel	All	High	No
Falbe et al,[83] 2015	Berkeley, US	$0.01 per ounce excise tax (SSBs)	Price changes	CBA	Primary: store audits	All	Medium	Yes
Falbe et al,[84] 2016	Berkeley, US	$0.01 per ounce excise tax (SSBs)	SSB consumption, consumption/ substitution	CBA	Primary: consumer surveys	Adults	Low	Yes
Falbe et al,[100] 2020	Oakland & San Francisco, US	$0.01 per ounce excise tax (SSBs)	Price changes	CBA	Primary: store audits	All	High	Yes
Fletcher et al,[95] 2010	US	0-7.25% sales tax (soft drinks)	BMI, dietary intake, SSB consumption, consumption/ substitution	Cross-sectional with fixed effects	Household survey: NHANES	Children	Medium	Yes
Fletcher et al,[97] 2010	US	0-7.25% sales tax (soft drinks)	BMI, SSB consumption	Cross-sectional	Household survey: NHANES	Children	Medium	Yes

© 2022 Andreyeva T et al. *JAMA Network Open.*

				with fixed effects				
Fletcher et al,[96] 2010	US	0-7.25% sales tax (soft drinks)	BMI	Cross-sectional with fixed effects	Household survey: BRFSS	Adults	Medium	Yes
Fletcher et al,[94] 2015	Ohio, US	$0.01 per 12-ounce can excise tax (soft drinks)<br><br>0-7.25% state sales tax (soft drinks)	BMI	CBA	Household survey: BRFSS	Adults	Medium	Yes
	US		BMI, dietary intake, SSB consumption, consumption/ substitution	Cross-sectional with fixed effects	Household survey: NHANES			
Goiana-da-Silva et al,[65] 2020	Portugal	€8.22 per hl (<80 g sugar/l) and €16.69 per hl (≥80 g sugar/l) excise tax (SSBs, ASBs)	Price changes, SSB sales	ITS with counterfactual	Scanner: Nielsen Consumer Panel	All	High	Yes
			Product change		Nutritional database: GlobalData			
Gonçalves and Santos ,[64] 2019	Portugal	€8.22 per hl (<80 g sugar/l) and €16.69 per hl (≥80 g sugar/l)	Price changes, SSB sales	Before and after with counterfactual	Scanner: one grocery chain	All	High	No[c]

© 2022 Andreyeva T et al. *JAMA Network Open.*

		excise tax (SSBs, ASBs)						
Grogger,[38] 2017	Mexico	1 peso per liter excise tax (SSBs)	Price changes	ITS with counterfactual	Administrative: INEGI	All	High	Yes
Guerrero-López et al,[43] 2017	Mexico	1 peso per liter excise tax (SSBs)	UC (unemployment)	Uncontrolled ITS	Industry survey: EMIM	All	High	Yes
Hashem et al,[49] 2019	United Kingdom	£0.24 per liter (≥8 g sugar/100 ml) and £0.18 per liter (5-8 g sugar/100 ml) excise tax (SSBs)	Product change	Uncontrolled before and after	Primary: product packaging audits Foodswitch United Kingdom	All	Low	Yes
Hoffer and Sheehan,[92] 2020	Washington, US	$0.02 per ounce excise tax (soft drinks)	SSB sales	CBA	Survey: Consumer expenditure surveys	All	Low	Yes
Law et al,[51] 2020	United Kingdom	£0.24 per liter (≥8 g sugar/100 ml) and £0.18 per liter (5-8 g sugar/100 ml) excise tax (SSBs)	UC (market return)	Uncontrolled ITS	Administrative: London Stock Exchange listed soft-drink firm reports	All	Low	Yes

© 2022 Andreyeva T et al. *JAMA Network Open.*

Law et al,[50] 2020	United Kingdom	£0.24 per liter (≥8 g sugar/100 ml) and £0.18 per liter (5-8 g sugar/100 ml) excise tax (SSBs)	UC (turnover)	Uncontrolled ITS	Industry survey: Monthly Business Survey	All	Medium	Yes
Lawman et al,[78] 2019	Philadelphia, US	$0.015 per ounce excise tax (SSBs, ASBs)	UC (unemployment)	Controlled ITS	Administrative: unemployment compensation program	All	High	Yes
Lee et al,[85] 2019	Berkeley, US	$0.01 per ounce excise tax (SSBs)	SSB consumption, consumption/ substitution	CBA	Primary: consumer surveys	Adults	Low	Yes
Leider et al,[103] 2018	Cook County, US	$0.01 per ounce excise tax (SSBs, ASBs)	Price changes	CBA	Primary: store audits	All	High	No
Lozano-Rojas,[86] 2020	Berkeley, US	$0.01 per ounce excise tax (SSBs)	Price changes, SSB sales	CBA	Scanner: Nielsen retail	All	High	No
Marinello et al,[101] 2019	Oakland, US	$0.01 per ounce excise tax (SSBs)	Price changes	CBA	Primary: restaurant audits	All	Medium	Yes

© 2022 Andreyeva T et al. *JAMA Network Open.*

Mendez,[87] 2019	Berkeley, US	$0.01 per ounce excise tax (SSBs)	Price changes, SSB sales	CBA	Scanner: Nielsen retail	All	High	No
Mora et al,[109] 2018	Catalonia, Spain	€0.08 per liter (5-8 g sugar/100 ml) and €0.12 per liter (≥8 g sugar/100 ml) excise tax (SSBs)	Price changes, SSB sales, sales/ substitution	CBA	Scanner: one grocery chain	All	High	No[c]
Nakamura et al,[59] 2018	Chile	10% (<6.25g sugar/100 ml) and 18% (≥6.25 g sugar/100ml) ad valorem excise tax (SSBs, ASBs)	Price changes, SSB sales, sales/ substitution	Uncontrolled ITS	Scanner: Kantar Worldpanel	All	High	Yes
Ng et al,[39] 2019	Mexico	1 peso per liter excise tax (SSBs)	SSB sales, sales/ substitution	Before and after with counterfactual	Scanner: Nielsen Mexico Consumer Panel Services	All	High	Yes
Oxford Economics,[79] 2017	Philadelphia, US	$0.015 per ounce excise tax (SSBs, ASBs)	Price changes, SSB sales, UC (cross-border)	Uncontrolled before and after	Scanner: IRI retail<br><br>Bottler sales	All	Low	No

© 2022 Andreyeva T et al. *JAMA Network Open.*

Pak,[98] 2013	US	0-7.25% state sales tax (soft drinks)	BMI	Cross-sectional with fixed effects	Household survey: BRFSS	Adults	Medium	No
Pedraza et al,[44] 2018	Mexico	1 peso per liter excise tax (SSBs)	SSB sales, sales/ substitution	Uncontrolled ITS	Scanner: Nielsen Mexico Consumer Panel Services	All	Medium	Yes
Pedraza et al,[40] 2019	Mexico	1 peso per liter excise tax (SSBs)	SSB sales, sales/ substitution	Uncontrolled ITS	Scanner: Nielsen Mexico Consumer Panel Services	All	High	Yes
Public Health England,[52] 2019	United Kingdom	£0.24 per liter (≥8 g sugar/100 ml) and £0.18 per liter (5-8 g sugar/100 ml) excise tax (SSBs)	Product change, SSB sales, sales/ substitution	Uncontrolled before and after	Scanner: Kantar Worldpanel	All	Medium	No
Public Health Seattle and King County,[108] 2019	Seattle, US	$0.0175 per ounce excise tax (SSBs)	Price changes	CBA	Primary: store audits	All	High	No
Pizzutti,[71] 2019	Philadelphia, US	$0.015 per ounce excise	SSB sales, sales/ substitution, UC (cross-border)	CBA	Scanner: one convenience store chain	All	High	No

© 2022 Andreyeva T et al. *JAMA Network Open.*

		tax (SSBs, ASBs)						
Powell et al,[104] 2020	Cook County, US	$0.01 per ounce excise tax (SSBs, ASBs)	SSB sales, sales/ substitution, UC (cross-border)	CBA	Scanner: Nielsen retail	All	High	Yes
Powell et al,[105] 2020	Cook County, US	$0.01 per ounce excise tax (SSBs, ASBs)	Price changes	CBA	Scanner: Nielsen retail	All	High	Yes
Powell and Leider,[106] 2020	Seattle, US	$0.0175 per ounce excise tax (SSBs)	Price changes, SSB sales, sales/ substitution, UC (cross-border)	CBA	Scanner: Nielsen retail	All	High	Yes
Roberto et al,[72] 2019	Philadelphia, US	$0.015 per ounce excise tax (SSBs, ASBs)	Price changes, SSB sales, sales/substitution, UC (cross-border)	CBA	Scanner: IRI retail	All	High	Yes
Rojas and Wang ,[88] 2017	Berkeley, US	$0.01 per ounce excise tax (SSBs)	Price changes, SSB sales, sales/ substitution	CBA	Scanner: Nielsen retail	All	High	No[c]
	Washington, US	$0.02 per 12-ounce can excise tax (carbonated soft drinks)						
Royo-Bordonada et al,[110] 2019	Catalonia, Spain	€0.08 per liter (5-8 g sugar/100	SSB consumption,	CBA	Primary: consumer survey	Adolescents, young adults	Low	Yes

© 2022 Andreyeva T et al. *JAMA Network Open.*

		ml) and €0.12 per liter (≥8 g sugar/100 ml) excise tax (SSBs)	consumption/ substitution					
Saelens et al,[107] 2020	Seattle, US	$0.0175 per ounce excise tax (SSBs)	Price changes	CBA	Primary: store audits	All	High	No
			SSB consumption, consumption/ substitution	CBA	Primary: longitudinal household survey	Adults, children	Low	
Sánchez-Romero et al,[45] 2020	Mexico	1 peso per liter excise tax (SSBs)	SSB consumption	Uncontrolled before and after	Survey: Health Workers Cohort Study	Adults	Low	Yes
Scarborough et al,[47] 2020	United Kingdom	£0.24per liter (≥8 g sugar/100 ml) and £0.18 per liter (5-8 g sugar/100 ml) excise tax (SSBs)	Price changes, product change	ITS with counterfactu al	Web-scaping: websites of 6 UK supermarkets using FoodDB; BrandView	All	High	Yes
Schmacker and Smed,[61] 2020	Denmark	0-Up to DKK 1.58 per liter (>0.5 g sugar/100 ml) and up to DKK 0.57	Price changes, SSB sales, sales/ substitution	Uncontrolled ITS	Scanner: GfK Denmark Consumer Panel	All	High	Yes

© 2022 Andreyeva T et al. *JAMA Network Open.*

		per liter (≤0.5 g sugar/100 ml) excise tax (SSBs, ASBs)						
Seiler et al,[73] 2019	Philadelphia, US	$0.015 per ounce excise tax (SSBs and ASBs)	Price changes, SSB sales, sales/ substitution, UC (cross-border)	CBA	Scanner: IRI retail	All	High	No[c]
Silver et al,[89] 2017	Berkeley, US	$0.01 per ounce excise tax (SSBs)	Price changes	Controlled ITS	Scanner: two grocery chains	All	High	Yes
				Uncontrolled before and after	Primary: store audits		Low	
			SSB sales, sales/ substitution, UC (cross-border)	Controlled ITS	Scanner: two grocery chains		High	
			SSB consumption, consumption/ substitution	Uncontrolled before and after	Primary: consumer survey	Adults	Low	
Stacey et al,[67] 2019	South Africa	0.021 ZAR (~$0.0015) per g sugar >4 g/100 ml excise tax (SSBs)	Price changes, product change	Uncontrolled ITS	Administrative: Statistics South Africa's Consumer Price Index	All	High	Yes

© 2022 Andreyeva T et al. *JAMA Network Open.*

Taylor et al,[90] 2019	Berkeley, US	$0.01 per ounce excise tax (SSBs)	SSB sales, sales/ substitution	Before and after with counterfactual	Scanner: university campus retail, Nielsen retail	Adults	Medium	Yes
Vall Castelló and Lopez Casasnovas,[111] 2020	Catalonia, Spain	€0.08 per liter (5-8 g sugar/100ml) and €0.12 per liter (≥8 g sugar/100ml) excise tax (SSBs)	Price changes	Uncontrolled before and after	Scanner: one supermarket chain	All	Low	Yes
			SSB sales, sales/ substitution	Uncontrolled ITS			Medium	
Zenk et al,[102] 2020	Oakland, US	$0.01 per ounce excise tax (SSBs)	UC (advertising)	CBA	Primary: store audits	All	Medium	Yes
Zhang and Palma,[91] 2020	Berkeley, US	$0.01 per ounce excise tax (SSBs)	Price changes	CBA	Scanner: Nielsen retail and Nielsen Consumer Panel	All	High	Yes
Zhong et al,[74] 2018	Philadelphia, US	$0.015 per ounce excise tax (SSBs, ASBs)	SSB consumption, consumption/ substitution	CBA	Primary: consumer survey	Adults	Low	Yes
Zhong et al,[75] 2020	Philadelphia, US	$0.015 per ounce excise tax (SSBs, ASBs)	SSB consumption, consumption/ substitution	CBA	Primary: consumer survey	Adults	Low	Yes

© 2022 Andreyeva T et al. *JAMA Network Open.*

Abbreviations: SSBs, sugar-sweetened beverages; ASBs, artificially-sweetened beverages; ITS, interrupted time series analysis; CBA, controlled before and after study; UC, unintended consequences; BMI, body mass index; INEGI, Mexico's Statistics Institute (*Instituto Nacional de Estadística y Geografía*, Spanish); ENIGH, National Income and Expenditure Household Survey (in Spanish); EMIM, Monthly Surveys of the Manufacturing Industry (in Spanish); NHANES, National Health and Nutrition Examination Survey; BRFSS, Behavioral Risk Factor Surveillance System.

Notes:
An ITS design is defined as a study design that uses longitudinal data and has three or more post-treatment periods. An ITS was further classified as a controlled ITS, ITS with a counterfactual, and uncontrolled ITS. A controlled ITS used non-treated sites as a comparison; an ITS with a counterfactual used a counterfactual that was not untreated sites, for example, the pre-treatment trend or untaxed beverages; an uncontrolled ITS did not have any type of comparison group. A before and after study used longitudinal data and made comparisons in two time periods: before and after tax implementation. Before and after studies were further classified as CBA, before and after with a counterfactual, uncontrolled before and after. The criteria for these classifications are the same as the ITS study designs. A cross-sectional study with fixed effects used repeated cross-sectional data and included fixed effects in their statistical model.

[a] Based on the study description;
[b] Not reporting #s of participants due to differences in the type of participants across studies (e.g., products, households, stores);
[c] Published in peer-reviewed literature since the end date of the review (June 1, 2020).

© 2022 Andreyeva T et al. *JAMA Network Open.*

eTable 3. Summary of Sensitivity and Sub-group Meta-Analyses

	Exclusion of Outlier Studies				Exclusion of Studies with Extreme Variance				Sub-Group of High Quality Studies			
Outcome	# studies	Pooled estimate (95% CI)	*P*-value	Heterogeneity: Q (*P*-value); I^2 % (95% CI)	# studies	Pooled estimate (95% CI)	*P*-value	Heterogeneity: Q (*P*-value); I^2 % (95% CI)	# studies	Pooled estimate (95% CI)	*P*-value	Heterogeneity Q (*P*-value); I^2 % (95% CI)
Price: tax pass through, %	33	84.4 (73.1 to 95.6)	<.001	225 (<.001); 85.8 (81.0 to 89.3)	N/A				32	78.7 (60.4 to 97.0)	<.001	5,087 (0); 99.4 (99.3 to 99.5)
SSB sales: % change in demand	17	-7.8 (-10.8 to -4.7)	<.001	304 (<.001); 94.7 (92.9 to 96.1)	33	-14.4 (-20.3 to -8.5)	<.001	13,112 (0); 99.8 (99.7 to 99.8)	22	-12.9 (-19.7 to -6.0)	<.001	709,041 (0); 100
SSB sales: price elasticity	20	-1.57 (-1.89 to -1.25)	<.001	1,126 (<.001); 98.3 (98.0-98.6)	33	-1.56 (-2.11 to -1.02)	<.001	8,194 (0); 99.6 (99.6; 99.6)	22	-1.39 (-1.86 to -0.91)	<.001	121,923 (0); 100
Sales, substitution: cross-price elasticity	23	0.28 (-0.27 to 0.83)	.30	980 (<.001); 97.8 (97.3 to 98.2)	23	0.38 (-0.56 to 1.32)	.41	370 (<.001); 94.1 (92.2 to 95.5)	17	0.24 (-0.25 to 0.73)	.32	955 (<.001); 98.3 (97.9 to 98.6)
SSB consumption: % change in demand	N/A				N/A				0			
SSB consumption: price elasticity	N/A				N/A				0			
Consumption, substitution: cross-price elasticity	10	0.06 (-0.10 to 0.22)	.42	4 (0.91) 0 (0 to 62.4)	N/A				0			

Abbreviations: SSB, sugar-sweetened beverages, CI, confidence interval, N/A, not available, no studies identified as outliers or studies with extreme variance.

© 2022 Andreyeva T et al. *JAMA Network Open.*

eTable 4. Sources of Heterogeneity (τ2) for 3-Level Random Effects Models		
	**Source of τ2 (95% CI)**	
**Outcome**	**Tax Jurisdiction**	**Study**
SSB consumption, price elasticity of taxed beverages	24.4 (6.3, 160.0)	< 0.0001 (0.0, 1.6)
SSB Sales, price elasticity of taxed beverages	0.2 (0.0, 1.5)	1.1 (0.5, 2.4)
Consumption of substitution beverages	0.8 (0.0, 11.3)	< 0.0001 (0.0, 0.41)
Indirect sales of substitution beverages	0.4 (0.0, 7.2)	3.4 (1.3, 8.7)
Tax pass through rate	0.07 (0.0, 0.2)	0.06 (0.0, 0.1)
SSB consumption,percent change in demand	0.03 (0.0, 0.23)	<0.0001 (0.0, 0.06)
SSB sales, percent change in demand	0.001 (0.001, 0.03)	0.004 (0.002, 0.01)

© 2022 Andreyeva T et al. *JAMA Network Open.*

eFigure 1. Meta-analysis of SSB Sales Following SSB Taxes: Percentage Change in Demand for Taxed Beverages

Source	Tax Jurisdiction	Estimate [95% CI]	Model Weight (%)
Alvarado et al,[63] 2019	Barbados	-0.04 [-0.05; -0.04]	6.7
Bollinger and Sexton,[82] 2018	Berkeley, US	-0.06 [-0.18; 0.06]	0.9
Lozano-Rojas,[86] 2020	Berkeley, US	-0.14 [-0.21; -0.08]	1.4
Mendez,[87] 2019	Berkeley, US	-0.00 [0.00; 0.00]	1.8
Rojas and Wang,[88] 2017	Berkeley, US	-0.01 [-0.07; 0.05]	1.4
Silver et al,[89] 2017	Berkeley, US	-0.10 [-0.10; -0.09]	1.8
Taylor et al,[90] 2019	Berkeley, US	-0.24 [-0.46; -0.02]	0.4
Taylor et al,[90] 2019	Berkeley, US	-0.12 [-0.16; -0.08]	1.6
Mora et al,[109] 2018	Catalonia, Spain	-0.01 [-0.01; 0.00]	4.3
Vall Castelló and Lopez Casasnovas,[111] 2020	Catalonia, Spain	-0.08 [-0.13; -0.03]	3.7
Caro et al,[57] 2018	Chile	-0.03 [-0.06; -0.01]	4.5
Nakamura et al,[59] 2018	Chile	-0.22 [-0.30; -0.13]	3.3
Powell et al,[104] 2020	Cook County, US	-0.27 [-0.30; -0.25]	6.7
Schmacker and Smed,[61] 2020	Denmark	-0.13 [-0.19; -0.07]	6.3
Capacci et al,[54] 2019	France	-0.06 [-0.23; 0.11]	4.1
Aguilar et al,[29] 2019	Mexico	-0.03 [-0.04; -0.01]	1.2
Arteaga et al,[30] 2017	Mexico	-0.04 [-0.05; -0.02]	1.2
Colchero et al,[34] 2016	Mexico	-0.06 [-0.09; -0.03]	1.2
Colchero et al,[33] 2016	Mexico	-0.07 [-0.10; -0.04]	1.2
Colchero et al,[35] 2017	Mexico	-0.10 [-0.10; -0.09]	1.2
Colchero et al,[37] 2017	Mexico	-0.06 [-0.07; -0.05]	1.2
Ng et al,[39] 2019	Mexico	-0.19 [-0.19; -0.19]	1.2
Pedraza et al,[40] 2019	Mexico	-0.19 [-0.24; -0.13]	1.1
Cawley et al,[99] 2020	Oakland, US	-0.59 [-1.44; 0.26]	0.4
Cawley et al,[69] 2019	Philadelphia, US	-0.30 [-0.46; -0.14]	2.7
Cawley et al,[70] 2019	Philadelphia, US	-0.62 [-1.21; -0.03]	0.3
Roberto et al,[72] 2019	Philadelphia, US	-0.35 [-0.66; -0.04]	1.0
Seiler et al,[73] 2019	Philadelphia, US	-0.46 [-0.62; -0.30]	2.7
Gonçalves and Santos,[64] 2019	Portugal	-0.05 [-0.22; 0.11]	4.2
Cornelsen et al,[114] 2017	Restaurant, UK	-0.09 [-0.15; -0.03]	6.3
Alsukait et al,[66] 2020	Saudi Arabia	-0.35 [-0.51; -0.18]	4.3
Powell and Leider,[106] 2020	Seattle, US	-0.22 [-0.25; -0.19]	6.6
Breeze et al,[113] 2018	Sheffield, UK	-0.27 [-0.33; -0.21]	6.2
Hoffer and Sheehan,[92] 2020	Washington, US	-0.89 [-1.76; -0.03]	0.1
Rojas and Wang,[88] 2017	Washington, US	-0.04 [-0.04; -0.04]	6.7
**Random effects model**		**-0.15 [-0.20; -0.09]**	**100.0**

I^2 = 100%, χ^2_{34} = 709741.71 (p < .001)
Test for overall effect: t_{34} = -5.12 (p < .001)

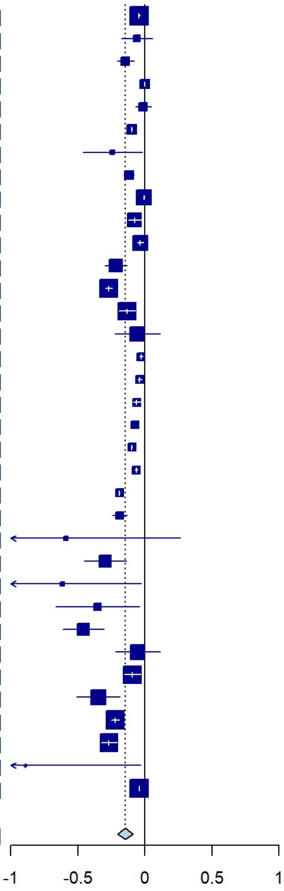

Abbreviations: SSBs, sugar-sweetened beverages.

© 2022 Andreyeva T et al. *JAMA Network Open.*

eFigure 2. Meta-analysis of Substitution Beverage Sales Following SSB Taxes: Cross-Price Elasticity

Source	Tax Jurisdiction	Elasticity [95% CI]	Model Weight (%)
Alvarado et al,[63] 2019	Barbados	0.88 [0.76; 1.00]	5.5
Rojas and Wang,[88] 2017	Berkeley, US	-0.92 [-3.30; 1.46]	3.6
Silver et al,[89] 2017	Berkeley, US	1.12 [0.97; 1.26]	5.1
Mora et al,[109] 2018	Catalonia, Spain	-0.51 [-0.87; -0.15]	5.0
Vall Castelló and Lopez Casasnovas,[111] 2020	Catalonia, Spain	8.04 [6.33; 9.75]	4.2
Caro et al,[57] 2018	Chile	-1.43 [-2.35; -0.51]	5.0
Nakamura et al,[59] 2018	Chile	-4.59 [-8.98; -0.19]	2.1
Powell et al,[104] 2020	Cook County, US	0.03 [-0.25; 0.31]	5.5
Schmacker and Smed,[61] 2020	Denmark	1.76 [0.38; 3.14]	4.9
Capacci et al,[54] 2019	France	-10.97 [-16.86; -5.07]	1.6
Colchero et al,[34] 2016	Mexico	0.42 [0.24; 0.60]	3.7
Colchero et al,[33] 2016	Mexico	0.56 [0.07; 1.05]	3.7
Colchero et al,[35] 2017	Mexico	-0.11 [-0.28; 0.07]	3.7
Colchero et al,[37] 2017	Mexico	1.72 [0.97; 2.47]	3.6
Ng et al,[39] 2019	Mexico	1.35 [1.35; 1.35]	3.7
Pedraza et al,[40] 2019	Mexico	1.22 [0.55; 1.89]	3.6
Cawley et al,[99] 2020	Oakland, US	3.62 [-7.41; 14.66]	0.6
Cawley et al,[69] 2019	Philadelphia, US	-0.47 [-1.25; 0.31]	4.3
Cawley et al,[70] 2019	Philadelphia, US	2.76 [-2.34; 7.86]	1.5
Roberto et al,[72] 2019	Philadelphia, US	0.02 [-0.24; 0.28]	4.5
Seiler et al,[73] 2019	Philadelphia, US	-0.15 [-0.63; 0.32]	4.4
Cornelsen et al,[114] 2017	Restaurant, UK	-1.85 [-3.67; -0.03]	4.5
Powell and Leider,[106] 2020	Seattle, US	0.26 [0.03; 0.49]	5.5
Breeze et al,[113] 2018	United Kingdom	2.43 [0.82; 4.04]	4.7
Rojas and Wang,[88] 2017	Washington, US	0.41 [0.10; 0.72]	5.5
**Random effects model**		**0.42 [-0.52; 1.35]**	**100.0**

I^2 = 98%, χ^2_{24} = 1056.02 (p < .001)
Test for overall effect: t_{24} = 0.92 (p = .37)

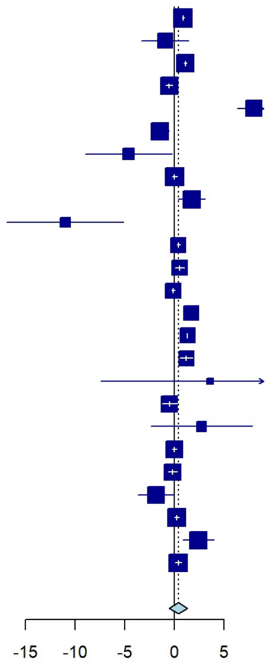

Abbreviations: SSBs, sugar-sweetened beverages.

© 2022 Andreyeva T et al. *JAMA Network Open.*

eFigure 3. Meta-analysis of SSB Consumption Following SSB Taxes: Price Elasticity of Demand for Taxed Beverages

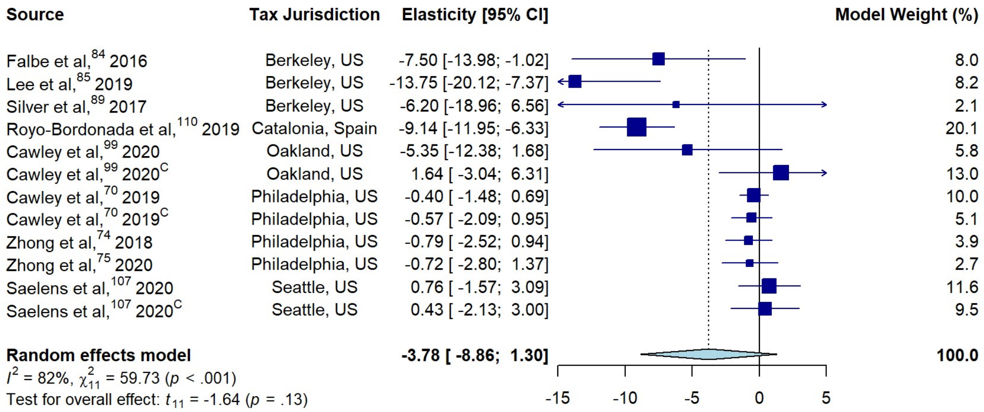

Abbreviations: SSBs, sugar-sweetened beverages.

© 2022 Andreyeva T et al. *JAMA Network Open.*

eFigure 4. Meta-analysis of SSB Consumption Following SSB Taxes: Percentage Change in Demand for Taxed Beverages

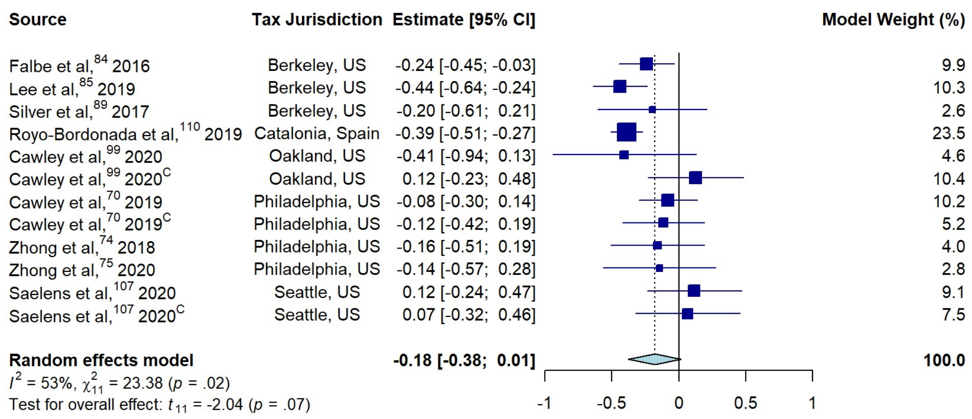

Abbreviations: SSBs, sugar-sweetened beverages.

© 2022 Andreyeva T et al. *JAMA Network Open.*

eFigure 5. Meta-analysis of Consumption of Substitution Beverages Following SSB Taxes: Cross-Price Elasticity

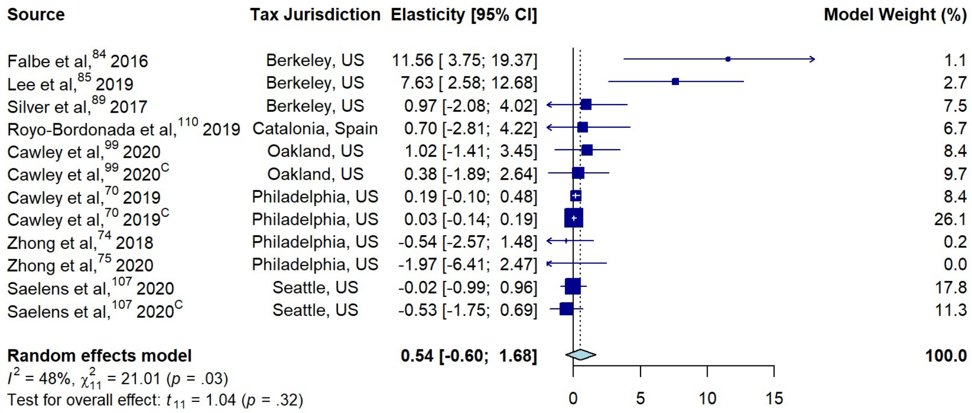

Abbreviations: SSBs, sugar-sweetened beverages.

© 2022 Andreyeva T et al. *JAMA Network Open.*

eFigure 6. Publication Bias: Funnel Plots

Price Effects: Tax Pass-Through RateEgger's test is not significant (*p*=0.88).

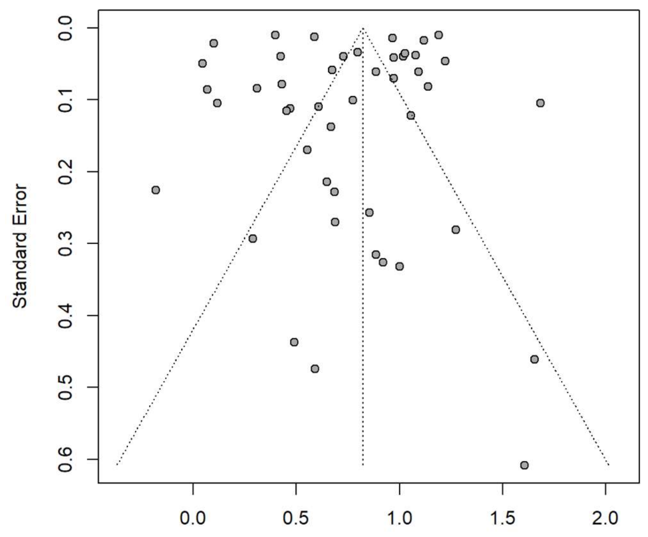

b) Sales of Substitution Beverages

- Eggers' test is significant (*p*<0.001).

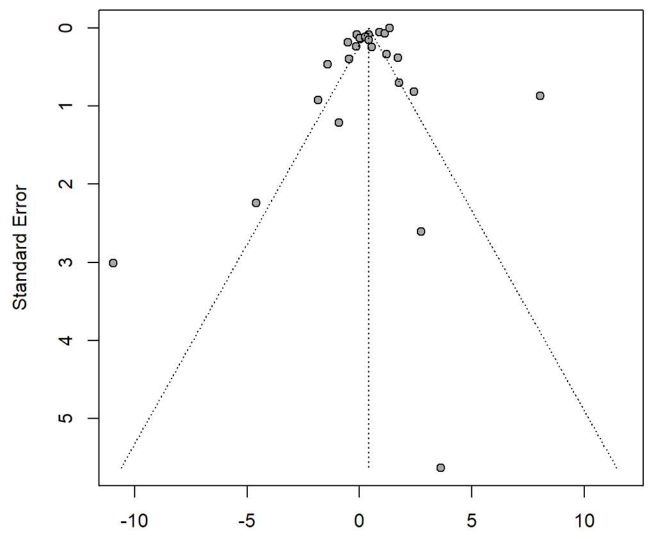

c) Consumption of Taxes Beverages: : Percent Change in Demand

© 2022 Andreyeva T et al. *JAMA Network Open.*

Egger’s test is significant (p=0.02).

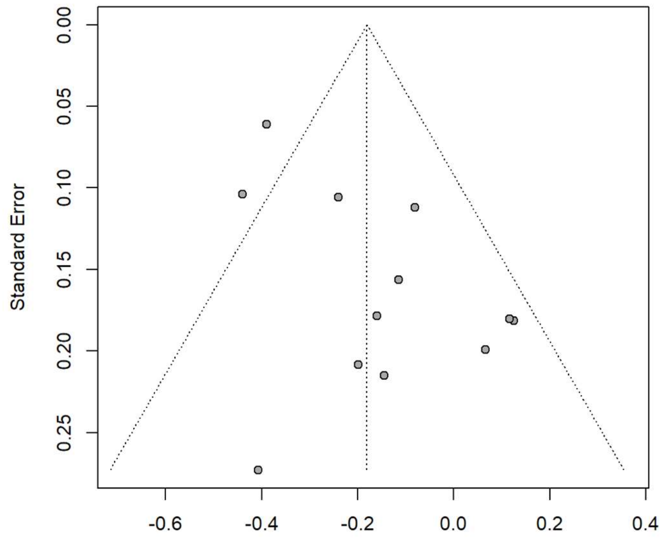

© 2022 Andreyeva T et al. *JAMA Network Open.*
